# Estimated glucose disposal rate outperforms other insulin resistance surrogates in predicting incident cardiovascular diseases in cardiovascular-kidney-metabolic syndrome stages 0–3 and the development of a machine learning prediction model: a nationwide prospective cohort study

**DOI:** 10.1186/s12933-025-02729-1

**Published:** 2025-04-16

**Authors:** Bingtian Dong, Yuping Chen, Xiaocen Yang, Zhengdong Chen, Hua Zhang, Yuan Gao, Enfa Zhao, Chaoxue Zhang

**Affiliations:** 1 Department of Ultrasound, The First Affiliated Hospital of Anhui Medical University, Hefei, 230022 China; 2 Liver Disease Center of Integrated Traditional Chinese and Western Medicine, Department of Radiology, Zhongda Hospital, Medical School, Southeast University, Nurturing Center of Jiangsu Province for State Laboratory of AI Imaging and Interventional Radiology (Southeast University), Nanjing, China; 3 Basic Medicine Research and Innovation Center of Ministry of Education, Zhongda Hospital, Southeast University, State Key Laboratory of Digital Medical Engineering, Nanjing, China; 4 Department of Ultrasound, Chenggong Hospital, Xiamen University, Xiamen, China; 5 Department of Internal Medicine, Diabetology and Nephrology, The Second Affiliated Hospital of Chongqing Medical University, Chongqing, 400010 China; 6 Department of Nephrology, The Second Affiliated Hospital of Chongqing Medical University, Chongqing, 400010 China; 7 Department of Ultrasound, Affiliated Hangzhou First People’s Hospital, School of Medicine, Westlake University, Hangzhou, China

**Keywords:** Cardiovascular-kidney-metabolic syndrome, Cardiovascular disease, Insulin resistance, Estimated glucose disposal rate

## Abstract

**Background:**

The American Heart Association recently introduced the concept of cardiovascular-kidney-metabolic (CKM) syndrome, highlighting the increasing importance of the complex interplay between metabolic, renal, and cardiovascular diseases (CVD). While substantial evidence supports a correlation between the estimated glucose disposal rate (eGDR) and CVD events, its predictive value compared with other insulin resistance (IR) indices, such as triglyceride–glucose (TyG) index, TyG-waist circumference, TyG-body mass index, TyG-waist-to-height ratio, triglyceride-to-high density lipoprotein cholesterol ratio, and the metabolic score for insulin resistance, remains unclear.

**Methods:**

This prospective cohort study utilized data from the China Health and Retirement Longitudinal Study (CHARLS). The individuals were categorized into four subgroups based on the quartiles of eGDR. The associations between eGDR and incident CVD were evaluated using multivariate logistic regression analyses and restricted cubic spline. Seven machine learning models were utilized to assess the predictive value of the eGDR index for CVD events. To assess the model’s performance, we applied receiver operating characteristic (ROC) and precision-recall (PR) curves, calibration curves, and decision curve analysis.

**Results:**

A total of 4,950 participants (mean age: 73.46 ± 9.93 years), including 50.4% females, were enrolled in the study. During follow-up between 2011 and 2018, 697 (14.1%) participants developed CVD, including 486 (9.8%) with heart disease and 263 (5.3%) with stroke. The eGDR index outperformed six other IR indices in predicting CVD events, demonstrating a significant and linear relationship with all outcomes. Each 1-unit increase in eGDR was associated with a 14%, 14%, and 19% lower risk of CVD, heart disease, and stroke, respectively, in the fully adjusted model. The incorporation of the eGDR index into predictive models significantly improved prediction performance for CVD events, with the area under the ROC and PR curves equal to or exceeding 0.90 in both the training and testing sets.

**Conclusions:**

The eGDR index outperforms six other IR indices in predicting CVD, heart disease, and stroke in individuals with CKM syndrome stages 0–3. Its incorporation into predictive models enhances risk stratification and may aid in the early identification of high-risk individuals in this population. Further studies are needed to validate these findings in external cohorts.

**Graphical abstract:**

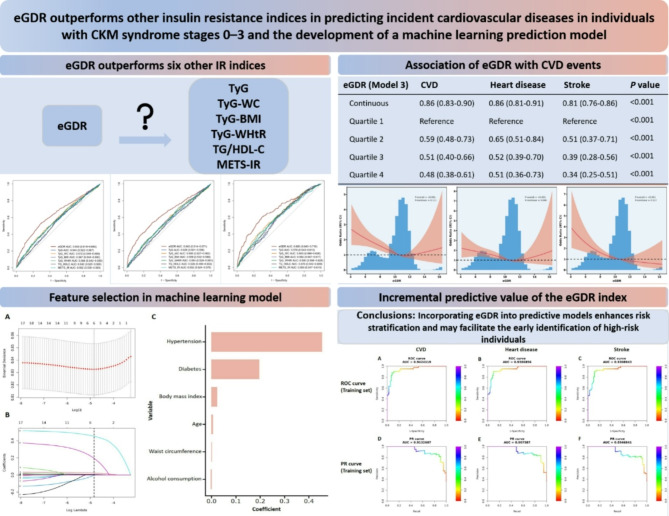

**Supplementary Information:**

The online version contains supplementary material available at 10.1186/s12933-025-02729-1.

## Research insights

**What is currently known about this topic?:**

Cardiovascular-kidney-metabolic (CKM) syndrome increases the risk of cardiovascular disease. Insulin resistance (IR) surrogate indices are associated with cardiovascular disease risk.

**What is the key research question?:**

How does estimated glucose disposal rate (eGDR) compare to other IR indices in predicting cardiovascular events?. Can machine learning achieve more precise risk stratification in the context of CKM syndrome?

**What is new?:**

eGDR outperforms six commonly used IR indices in predicting cardiovascular events.Machine learning models incorporating eGDR improve predictive value, enhancing risk stratification.This is the first large-scale study to validate eGDR’s superiority in CKM syndrome.

**How might this study influence clinical practice?:**

Findings could improve early identification of high-risk individuals in CKM syndrome.

**Supplementary Information:**

The online version contains supplementary material available at 10.1186/s12933-025-02729-1.

## Introduction

In October 2023, the American Heart Association (AHA) issued a Presidential Advisory defining cardiovascular-kidney-metabolic (CKM) syndrome as a systemic disorder resulting from complex interactions among metabolic risk factors, chronic kidney disease (CKD), and the cardiovascular system [1]. CKM syndrome represents an interconnected spectrum of conditions, wherein metabolic abnormalities, CKD, and cardiovascular diseases (CVD) synergistically elevate the risk of multiorgan dysfunction and adverse cardiovascular outcomes [1, 2]. Specifically, patients with heart failure (HF) have a four-fold higher prevalence of type 2 diabetes (T2D) (20%) compared to those without HF (4**–**6%) [3]. Additionally, T2D is associated with a two- to four-fold increased risk of CVD [4], while CKD affects nearly 40% of individuals with T2D and 50% of those with HF [2, 5].

In the United States, more than 25% of adults suffered from cardiac, renal, and metabolic diseases between 2015 and 2020 [6]. The intricate interplay among the cardiovascular, renal, and metabolic systems emphasizes the critical need for strategies to mitigate CKM syndrome’s burden [1, 2]. The AHA stresses the critical need for early screening of individuals in stages 0 to 3 of CKM syndrome, particularly to prevent CVD events [1]. Substantial evidence suggests that the clinical burden of CKM syndrome is predominantly driven by CVD [1, 2, 7, 8], highlighting the necessity of addressing the metabolic, renal, and cardiovascular components as an integrated system. This approach is essential not only to prevent disease progression across stages 0–3 but also to identify reliable biomarkers that can improve risk stratification, guide therapeutic decisions, and ultimately optimize patient outcomes.

Among the multifaceted mechanisms underlying CKM syndrome, insulin resistance (IR) plays a pivotal role as a key driver of metabolic dysfunction [1]. It promotes atherosclerosis, renal impairment, and systemic inflammation and serves as an independent risk factor for adverse cardiovascular outcomes. In this context, the estimated glucose disposal rate (eGDR) has emerged as a validated and practical surrogate marker for quantifying IR. Derived from clinical parameters such as waist circumference (WC), hypertension status, and glycosylated hemoglobin A1c (HbA1c), eGDR provides a novel and noninvasive measure of insulin sensitivity [9]. Previous studies have demonstrated its strong correlation with metabolic and cardiovascular risks, particularly in populations with T2D [9–16]. Moreover, eGDR has shown superior predictive value for CVD incidence and mortality compared to other IR indices, such as the triglyceride–glucose (TyG) index and TyG-derived parameters [10, 14–16]. Despite these promising findings, its comparative utility in the broader CKM syndrome population remains underexplored, suggesting the need for further investigation.

With the rapid advancement of big data and computational technology, machine learning has become a powerful tool in medical research, particularly for disease risk prediction and personalized treatment in patients with metabolic diseases at increased cardiovascular risk [17, 18]. Traditional statistical methods often struggle to handle complex, multidimensional clinical data, whereas machine learning algorithms can uncover hidden patterns within large datasets, enabling more accurate predictions [17, 18]. However, the potential of machine learning models to assess the relationship between eGDR and incident CVD in populations with CKM syndrome has yet to be fully explored.

Given these lines of evidence, we aimed to evaluate the association between eGDR and the incidence of CVD in individuals with CKM syndrome using machine learning algorithms. Additionally, we sought to compare the predictive value of eGDR against several other IR indices, including TyG, TyG-WC, TyG-body mass index (TyG-BMI), TyG-waist-to-height ratio (TyG-WHtR), triglyceride (TG)-to-high density lipoprotein cholesterol (HDL-C) ratio (TG/HDL-C), and the metabolic score for insulin resistance (METS-IR). By focusing on this high-risk population and addressing existing knowledge gaps, this study aims to develop a reliable tool for risk assessment, facilitating better stratification and enabling timely interventions to improve clinical outcomes.

## Methods

### Study design and population

We extracted data from the China Health and Retirement Longitudinal Study (CHARLS), which includes Chinese adults aged 45 years and older. The study design and inclusion criteria have been extensively described in previous publications [19]. Briefly, the dataset encompasses baseline and follow-up data, collected through structured interviews and clinical measurements, covering a wide range of socio-demographic, health-related, and lifestyle factors. The study adhered to the principles of the Declaration of Helsinki and received approval from the Biomedical Ethics Review Board of Peking University (IRB 00001052–11015). Written informed consent was obtained from all participants prior to their inclusion in the study. Further details about CHARLS are available on its official website (http://charls.pku.edu.cn/en).

The CHARLS national baseline survey was conducted from June 2011 to March 2012, with participants undergoing regular follow-ups every two years through face-to-face interviews. These interviews were conducted by trained interviewers using computer-assisted techniques to ensure standardized data collection [18]. In this study, participants who were interviewed between 2011 and 2012 were considered part of the baseline cohort, with follow-up data collected in 2013, 2015, and 2018.

**Fig. 1:**
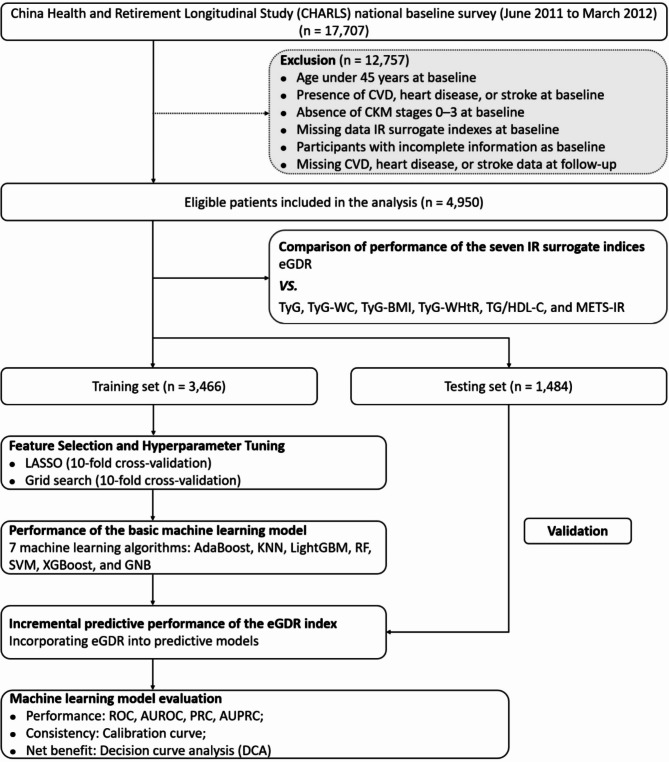
Flowchart of the study

The inclusion and exclusion criteria for this study are depicted in the flowchart (Fig. 1). Of the 17,707 participants from the 2011 baseline survey, 12,757 participants were excluded for the following reasons: (1) age under 45 years at baseline; (2) presence of CVD, heart disease, or stroke at baseline; (3) absence of CKM stages 0–3 at baseline; (4) missing data for one of the seven IR surrogate indexes at baseline; (5) incomplete information on anthropometric, health-related, sociodemographic, or other biomarkers at baseline; and (6) missing CVD, heart disease, and stroke data at follow-up. As a result, 4,950 participants were included in the final analysis.

### Definition of IR surrogate indices

IR was evaluated using several validated surrogate indices, derived from easily accessible clinical parameters. The primary index used was the eGDR index, which was calculated based on WC, hypertension status, and HbA1c levels. Additionally, for comparative purposes, six other commonly used IR indices were included. The eGDR index and other IR indices were calculated following the methods described in previous studies [9, 15], and the detailed calculation procedures are provided in the Supplementary Materials and Methods, Part I.

### Definition of CKM syndrome stages 0 to 4

According to the AHA Presidential Advisory Statement [1], the stages of CKM syndrome are defined as follows: Stage 0: No CKM risk factors. Stage 1: Excess or dysfunctional adiposity. Stage 2: Metabolic disorders (such as T2D, hypertension, and high triglycerides) or CKD. Stage 3: Subclinical CVD within the context of CKM syndrome. Stage 4: Clinical CVD, including conditions like coronary heart disease, HF, stroke, peripheral artery disease, and atrial fibrillation, in the setting of CKM.

### Ascertainment of outcomes

The primary outcome of interest was the incidence of CVD, including heart disease and stroke, as diagnosed based on self-reports. Participants confirmed having received a definitive diagnosis of CVD from their physicians, consistent with established precedents [20, 21]. Incident CVD events were defined as new-onset cases that occurred during the follow-up period, from baseline (2011) to the most recent available follow-up data (2018), whichever came first. The CHARLS study team implemented strict quality control measures to ensure data accuracy and reliability [19].

### Data collection

The CHARLS investigators collected variables according to pre-specified standards. The following data from the baseline survey were collected for this study: (1) Demographic data: age, gender, education level, and marital status; (2) Body measurements: systolic blood pressure (SBP), diastolic blood pressure (DBP), and WC; (3) Lifestyle data: smoking and alcohol consumption status; (4) Disease status: hypertension and diabetes; and (5) Laboratory test data: TG, total cholesterol (TC), HDL-C, low density lipoprotein cholesterol (LDL-C), serum creatinine (Scr), fasting blood glucose (FBG), HbA1c, and uric acid (UA). IR surrogate indices were assessed through further calculations.

Participants’ blood pressure was calculated as the average of three measurements taken while seated after resting for five minutes. Hypertension was defined as a self-reported diagnosis by a physician, use of antihypertensive medications, or an SBP of ≥ 130 mmHg or DBP of ≥ 80 mmHg [22]. Diabetes was defined as a self-reported diagnosis by a physician, use of hypoglycemic drugs, FBG ≥ 7.0 mmol/L (126 mg/dL), and/or an HbA1c level ≥ 6.5% at baseline [23].

### Model development and validation

Feature selection was performed using the least absolute shrinkage and selection operator (LASSO) algorithm [24], which effectively performs both variable selection and regularization. This approach improves model interpretability and helps prevent overfitting by shrinking less important variables to zero. Initially, the selected variables were used to develop basic predictive models for CVD risk. The dataset was randomly divided into training and testing sets in a 7:3 ratio for model development and validation.

Seven machine learning models were trained on the training set to predict the risk of incident CVD: Adaptive Boosting (AdaBoost), K-Nearest Neighbor (KNN), Light Gradient Boosting Machine (LightGBM), Random Forest (RF), Support Vector Machine (SVM), eXtreme Gradient Boosting (XGBoost), and Gaussian Naive Bayes (GNB), were trained on the training cohort to predict the risk of incident CVD. A brief description of these machine learning algorithms is provided in the Supplementary Materials and Methods, Part II. Hyperparameter tuning was performed using grid search technique, with optimization of model performance based on 10-fold cross-validation.

### Statistical analysis

Continuous variables were presented as means with standard deviations or medians with interquartile ranges, depending on their distribution. Categorical variables were expressed as frequencies and percentages. Comparisons between groups were made using the independent *t*-test or one-way analysis of variance (ANOVA) for normally distributed continuous variables, and the Mann-Whitney *U* test for non-normally distributed continuous variables. Categorical variables were compared using the chi-square test. Missing data were handled using multiple imputations to ensure the robustness of the results.

Three logistic regression models were constructed with varying levels of adjustment: (1) Model 1 was unadjusted; (2) Model 2 was adjusted for age, gender, education level, marital status, smoking status, and alcohol consumption status; and (3) Model 3 included adjustments for age, BMI, WC, hypertension, diabetes, and alcohol consumption status, with these variables selected using the LASSO algorithm. To assess potential multicollinearity among the variables in each model, we used the variance inflation factor (VIF). The VIF values for all variables were below 5, indicating no significant multicollinearity issues. To investigate the dose–response relationship between eGDR and the incidence of CVD, restricted cubic splines (RCS) based on logistic regression models were employed. We fitted RCS models with 3 to 5 nodes and selected the model with the lowest Akaike information criterion (AIC) to determine the optimal number of nodes. Subgroup and interaction analyses were performed by stratifying and clustering by age, gender, BMI, hypertension status, diabetes status, and others to examine the variations in the association between eGDR and CVD likelihood across different subgroups.

### Comparison of performance of the seven IR surrogate indices

We compared the performance of eGDR with six other IR indices (TyG, TyG-WC, TyG-BMI, TyG-WHtR, TG/HDL-C, and METS-IR) for predicting CVD heart disease, and stroke using several key metrics. These included: receiver operating characteristic (ROC) curves, area under the ROC curve (AUC), sensitivity, specificity, positive predictive value, and negative predictive value. The DeLong test was employed to compare the AUCs of the indices.

### Performance of the basic machine learning model

The performance of the basic machine learning model was assessed using ROC curves, AUC, sensitivity, specificity, accuracy, and F1-score. The DeLong test was used to compare differences between various AUCs. The best-performing machine learning algorithm was applied to evaluate the performance of the basic model and an optimized model, which incorporated the eGDR index, by comparing their concordance statistics (AUC).

### Incremental predictive performance of the eGDR index

In addition to the ROC curve, the area under the precision-recall curve (AUPRC) was calculated for predicting the incidence of CVD events, particularly useful for imbalanced datasets. Unlike AUC, AUPRC focuses on the model’s ability to predict the positive class, combining precision and recall. Decision curve analysis (DCA) and calibration curves were used to further assess and validate the final models’ performance. The calibration of clinical prediction models was evaluated using the Hosmer-Lemeshow test, with a *P* value > 0.05 indicating a good fit between the model and the actual data.

Statistical analyses were conducted using R (version 4.2.1, R Foundation) and IBM SPSS (version 26.0, IBM, Armonk, NY, USA). A two-sided *P* value of < 0.05 was considered statistically significant. Machine learning models were developed using the Python Scikit-learn library (version 1.1.3, https://github.com/scikit-learn/scikit-learn).

## Results

### Baseline characteristics

A total of 4,950 participants (mean age: 73.46 ± 9.93 years), including 50.4% females, were enrolled in the study. Supplementary Fig. 1 shows the distribution of the eGDR, with a mean value of 9.98 ± 2.02. The distributions of the eGDR index for CVD, heart disease, and stroke are presented in Supplementary Fig. 2. The baseline characteristics stratified by quartiles of eGDR (Q1: <9.08; Q2: 9.08–10.53; Q3: 10.53–11.31; Q4: >11.31) are presented in Table 1. In brief, SBP, DBP, BMI, WC, TG, TC, Scr, FPG, HbA1c, UA, the proportion of diabetes patients, and the incidence of CVD, heart disease, and stroke, as well as TyG, TyG-WC, TyG-BMI, TyG-WHtR, TG/HDL-C, and METS-IR, all decreased with increasing eGDR (all *P* < 0.001). However, individuals with higher eGDR levels tended to have a higher proportion of smoking and alcohol consumption (all *P* < 0.001).

**Table 1:** Baseline characteristics and CVD events documented during follow-up of the study population stratified by quartiles of eGDR

Characteristics	Total (*n* = 4950)	Quartiles of eGDR				*P* value
		Quartile 1 (*n* = 1237)	Quartile 2 (*n* = 1237)	Quartile 3 (*n* = 1239)	Quartile 4 (*n* = 1237)	
Age (years)	73.46 ± 9.929	75.16 ± 9.433	72.46 ± 9.567	72.93 ± 10.193	73.32 ± 10.292	< 0.001
Female, n (%)	2,495 (50.4%)	670 (54.2%)	685 (55.4%)	585 (47.2%)	555 (44.9%)	< 0.001
*Education level,* n (%)						0.022
Below primary school	1,324 (26.7%)	350 (28.3%)	306 (24.7%)	325 (26.2%)	343 (27.7%)	
Primary school	2,139 (43.2%)	531 (42.9%)	505 (40.8%)	548 (44.2%)	555 (44.9%)	
Middle school	1,016 (20.5%)	249 (20.1%)	282 (22.8%)	252 (20.3%)	233 (18.8%)	
High school or above	471 (9.5%)	107 (8.6%)	144 (11.6%)	114 (9.2%)	106 (8.6%)	
*Marital status*, n (%)						0.755
Married	4,754 (96.0%)	1,186 (95.9%)	1,184 (95.7%)	1,190 (96.0%)	1,194 (96.5%)	
Others	196 (4.0%)	51 (4.1%)	53 (4.3%)	49 (4.0%)	43 (3.5%)	
SBP (mmHg)	130.30 ± 21.511	143.18 ± 22.081	129.83 ± 19.692	125.32 ± 18.931	122.87 ± 19.291	< 0.001
DBP (mmHg)	75.55 ± 12.090	81.41 ± 12.508	76.16 ± 11.245	73.07 ± 11.022	71.57 ± 11.127	< 0.001
BMI (kg/m^2^)	23.36 ± 3.870	25.47 ± 4.374	25.19 ± 2.862	22.45 ± 2.655	20.36 ± 2.822	< 0.001
WC (cm)	83.94 ± 12.247	91.12 ± 10.734	91.63 ± 5.313	82.34 ± 3.364	70.68 ± 12.514	< 0.001
TG (mg/dL)	127.99 ± 90.416	150.05 ± 108.374	142.13 ± 99.444	118.78 ± 77.493	101.01 ± 59.860	< 0.001
TC (mg/dL)	193.67 ± 37.826	197.53 ± 38.774	197.64 ± 38.200	193.08 ± 36.976	186.43 ± 36.242	< 0.001
HDL-C (mg/dL)	51.86 ± 15.375	48.25 ± 14.935	48.86 ± 14.642	53.13 ± 14.600	57.18 ± 15.607	< 0.001
LDL-C (mg/dL)	116.89 ± 34.715	119.50 ± 36.776	120.11 ± 35.330	117.47 ± 33.896	110.47 ± 31.863	< 0.001
Scr (mg/dl)	0.79 ± 0.266	0.82 ± 0.372	0.78 ± 0.234	0.78 ± 0.183	0.78 ± 0.235	< 0.001
FPG (mg/dL)	109.28 ± 34.858	122.77 ± 55.825	109.38 ± 27.722	103.16 ± 18.014	101.80 ± 19.482	< 0.001
HbA1c (%)	5.25 ± 0.767	5.59 ± 1.208	5.31 ± 0.586	5.13 ± 0.410	4.97 ± 0.411	< 0.001
UA, mg/dL	4.48 ± 1.259	4.73 ± 1.346	4.55 ± 1.251	4.34 ± 1.201	4.30 ± 1.187	< 0.001
Smoking, n (%)	2,042 (41.3%)	467 (37.8%)	447 (36.1%)	534 (43.1%)	594 (48.0%)	< 0.001
Alcohol consumption, n (%)	1,721 (34.8%)	378 (30.6%)	408 (33.0%)	456 (36.8%)	479 (38.7%)	< 0.001
Hypertension, n (%)	1,077 (21.8%)	1,042 (84.2%)	7 (0.6%)	6 (0.5%)	22 (1.8%)	< 0.001
Diabetes, n (%)	229 (4.6%)	142 (11.5%)	51 (4.1%)	23 (1.9%)	13 (1.1%)	< 0.001
Heart disease, n (%)	486 (9.8%)	182 (14.7%)	119 (9.6%)	93 (7.5%)	92 (7.4%)	< 0.001
Stroke, n (%)	263 (5.3%)	115 (9.3%)	58 (4.7%)	47 (3.8%)	43 (3.5%)	< 0.001
CVD, n (%)	697 (14.1%)	270 (21.8%)	168 (13.6%)	129 (10.4%)	130 (10.5%)	< 0.001
*IR surrogate indices*						
eGDR	9.98 ± 2.021	7.01 ± 1.170	9.97 ± 0.399	10.92 ± 0.219	12.00 ± 0.976	< 0.001
TyG	8.66 ± 0.639	8.90 ± 0.711	8.77 ± 0.619	8.56 ± 0.566	8.41 ± 0.528	< 0.001
TyG-WC	728.77 ± 131.376	812.76 ± 128.774	803.98 ± 75.917	705.03 ± 54.705	593.35 ± 110.116	< 0.001
TyG-BMI	202.99 ± 40.784	227.23 ± 46.309	221.11 ± 30.863	192.34 ± 27.111	171.31 ± 28.146	< 0.001
TyG-WHtR	4.62 ± 0.846	5.14 ± 0.800	5.09 ± 0.539	4.47 ± 0.441	3.78 ± 0.726	< 0.001
TG/HDL-C	3.07 ± 4.163	3.93 ± 5.516	3.60 ± 4.953	2.67 ± 2.903	2.07 ± 1.950	< 0.001
METS-IR	35.21 ± 8.360	39.91 ± 9.591	38.70 ± 7.131	33.09 ± 5.521	29.16 ± 5.579	< 0.001

*eGDR*, estimated glucose disposal rate; *SBP*, systolic blood pressure; *DBP*, diastolic blood pressure; *BMI*, body mass index; *WC*, waist circumference; *TG*, triglyceride; *TC*, total cholesterol; *HDL-C*, high-density lipoprotein cholesterol; *LDL-C*, low-density lipoprotein cholesterol; *Scr*, serum creatinine; *FPG*, fasting plasma glucose; *HbA1c*, glycosylated hemoglobin A1c; *UA*, uric acid; *IR*, insulin resistance; *TyG*, triglyceride–glucose; *TyG-WC*, TyG-waist circumference; *TyG-BMI*, TyG-body mass index; *TyG-WHtR*, TyG-waist-to-height ratio; *TG/HDL-C*, triglyceride-to-high density lipoprotein cholesterol ratio; *METS-IR*, metabolic score for insulin resistance

Data are presented as mean ± standard deviation or number (%)

During follow-up between 2011 and 2018, 697 (14.1%) participants developed CVD, including 486 (9.8%) with heart disease and 263 (5.3%) with stroke. The comparisons of baseline characteristics between those with and without CVD, heart disease, and stroke are described in Supplementary Tables 1, 2 and 3.

### Predictive value of eGDR and other IR indices for the incidence of CVD

**Fig. 2:**
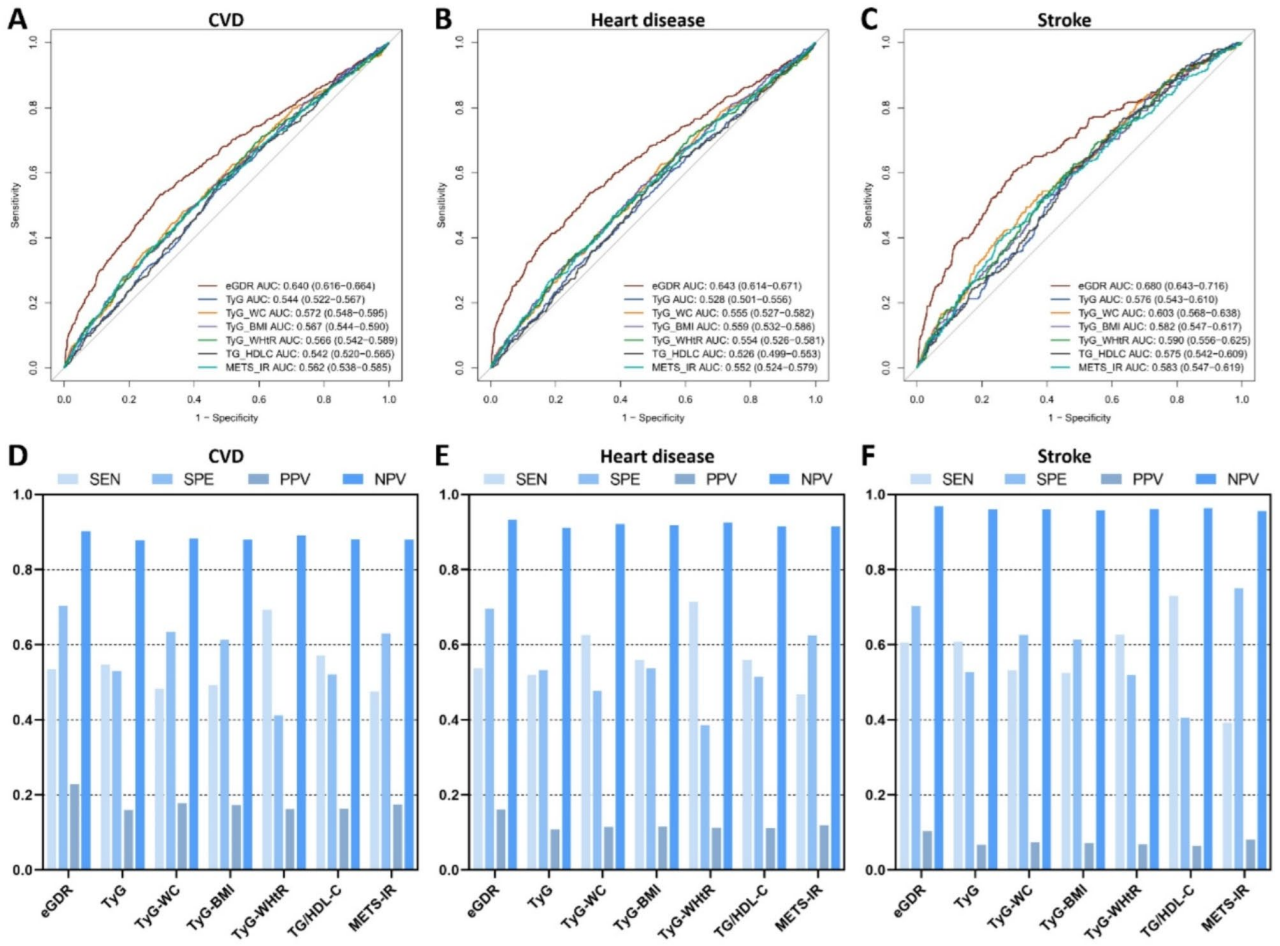
Predictive value of seven IR surrogate indices for cardiovascular diseases in individuals with cardiovascular-kidney-metabolic syndrome stages 0–3. *CVD*, cardiovascular disease; *IR*, insulin resistance; *SEN*, sensitivity; *SPE*, specificity; *PPV*, positive predictive value; *NPV*, negative predictive value; *eGDR*, estimated glucose disposal rate; *TyG*, triglyceride–glucose; *TyG-WC*, TyG-waist circumference; *TyG-BMI*, TyG-body mass index; *TyG-WHtR*, TyG-waist-to-height ratio; *TG/HDL-C*, triglyceride-to-high density lipoprotein cholesterol ratio; *METS-IR*, metabolic score for insulin resistance

The performance of seven IR surrogate indices, including eGDR, TyG, TyG-WC, TyG-BMI, TyG-WHtR, TG/HDL-C, and METS-IR, for predicting CVD, heart disease, and stroke is shown in Fig. 2. We found that eGDR had the highest AUC values for predicting CVD (0.640, 95% confidence interval [CI]: 0.616–0.664), heart disease (0.643, 95% CI 0.614–0.671), and stroke (0.680, 95% CI 0.643–0.716). When comparing the predictive abilities of the different IR indices, eGDR outperformed the other indices in predicting CVD, heart disease, and stroke (all *P* < 0.05). As a result, we selected eGDR as the best IR index for further analysis.

### Associations of baseline eGDR with incident CVD

**Fig. 3:**
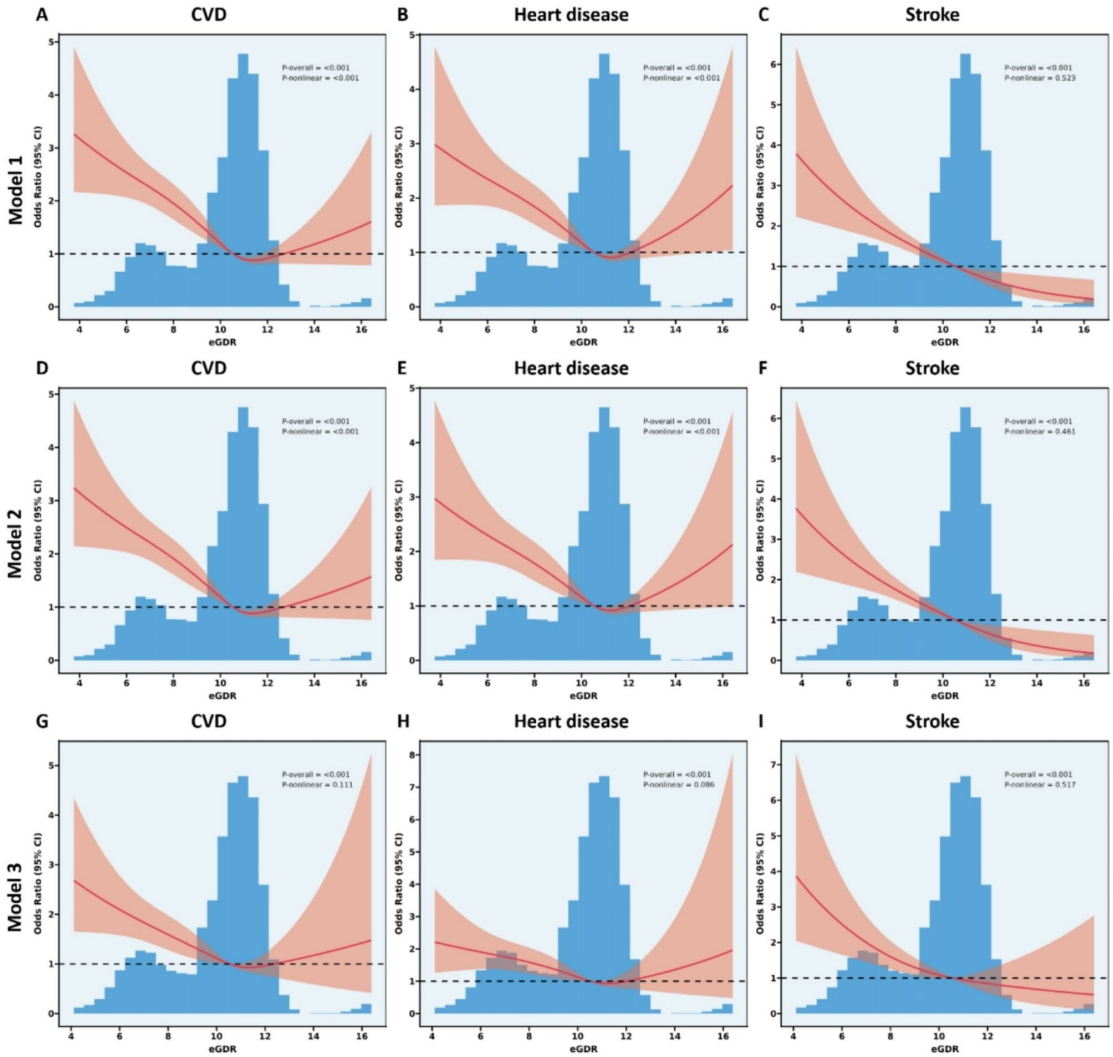
Restricted cubic spline curves for CVD, heart disease, and stroke according to the eGDR in the **A**,** B**,**and C** Model 1, **D**,** E**,**and F** Model 2, and **G**,** H**,**and I** Model 3, respectively. Model 1 was unadjusted; Model 2 was adjusted for age, gender, education level, marital status, smoking status, and alcohol consumption status; and Model 3 adjusted age, BMI, WC, hypertension, diabetes, and alcohol consumption status. *CVD*, cardiovascular disease; *eGDR*, estimated glucose disposal rate; *BMI*, body mass index; *WC*, waist circumference; *OR*, odds ratio

The dose–response curves between eGDR and the incidence of CVD, heart disease, and stroke are presented in Fig. 3. These RCS curves demonstrated a significant and linear relationship between eGDR and the incidence of all three outcomes, with full adjustment for covariates in Model 3 (all *P* for overall < 0.001 and *P* for non-linear > 0.05). A linear relationship between eGDR and the incidence of stroke was observed both with and without covariate adjustment (all *P* for overall < 0.001 and *P* for non-linear > 0.05). The RCS model showed non-linear dose–response associations between eGDR and the risk of CVD and heart disease in Models 1 and 2 (all *P* for overall < 0.001 and *P* for non-linear < 0.001).

The unadjusted and multivariate-adjusted odds ratios (ORs) and 95% CIs of eGDR for CVD, heart disease, and stroke are provided in Table 2. Compared to participants in the lowest quartile of eGDR, those in the highest quartile had a lower risk of CVD, heart disease, and stroke in the unadjusted model (Model 1). Similarly, after adjusting for age, gender, education level, marital status, smoking, and alcohol consumption (Model 2), the association between eGDR and the risks of these outcomes remained significant. In the fully adjusted model (Model 3), participants in the highest eGDR quartile had a 52% lower risk of CVD (OR: 0.48, 95% CI 0.38–0.61), a 49% lower risk of heart disease (OR: 0.51, 95% CI 0.36–0.73), and a 66% lower risk of stroke (OR: 0.34, 95% CI 0.25–0.51) compared to those in the lowest quartile. When eGDR was analyzed as a continuous variable, each 1-unit increase in eGDR was associated with a 14%, 14%, and 19% lower risk of CVD, heart disease, and stroke, respectively, in the fully adjusted model.

**Table 2:** Multivariate regression analysis of the associations between eGDR and cardiovascular diseases in individuals with CKM syndrome stages 0–3

eGDR	Model 1		Model 2		Model 3	
	OR (95%CI)	*P* value	OR (95%CI)	*P* value	OR (95%CI)	*P* value
*Cardiovascular diseases*						
Continuous	0.84 (0.81–0.87)	< 0.001	0.84 (0.81–0.88)	< 0.001	0.86 (0.83–0.90)	< 0.001
Quartile 1	Reference		Reference		Reference	
Quartile 2	0.56 (0.46–0.70)	< 0.001	0.58 (0.47–0.72)	< 0.001	0.59 (0.48–0.73)	< 0.001
Quartile 3	0.42 (0.33–0.52)	< 0.001	0.44 (0.34–0.54)	< 0.001	0.51 (0.40–0.66)	< 0.001
Quartile 4	0.41 (0.34–0.53)	< 0.001	0.43 (0.34–0.54)	< 0.001	0.48 (0.38–0.61)	< 0.001
*P* for trend	< 0.001		< 0.001		< 0.001	
*Heart disease*						
Continuous	0.86 (0.83–0.90)	< 0.001	0.87 (0.83–0.91)	< 0.001	0.86 (0.81–0.91)	< 0.001
Quartile 1	Reference		Reference		Reference	
Quartile 2	0.62 (0.48–0.79)	< 0.001	0.63 (0.49–0.80)	< 0.001	0.65 (0.51–0.84)	< 0.001
Quartile 3	0.48 (0.36–0.61)	< 0.001	0.49 (0.37–0.63)	< 0.001	0.52 (0.39–0.70)	< 0.001
Quartile 4	0.47 (0.36–0.61)	< 0.001	0.48 (0.37–0.63)	< 0.001	0.51 (0.36–0.73)	< 0.001
*P* for trend	< 0.001		< 0.001		< 0.001	
*Stroke*						
Continuous	0.81 (0.76–0.85)	< 0.001	0.81 (0.76–0.85)	< 0.001	0.81 (0.76–0.86)	< 0.001
Quartile 1	Reference		Reference		Reference	
Quartile 2	0.48 (0.35–0.67)	< 0.001	0.50 (0.36–0.70)	< 0.001	0.51 (0.37–0.71)	< 0.001
Quartile 3	0.39 (0.27–0.55)	< 0.001	0.39 (0.27–0.55)	< 0.001	0.39 (0.28–0.56)	< 0.001
Quartile 4	0.35 (0.25–0.50)	< 0.001	0.35 (0.24–0.50)	< 0.001	0.34 (0.25–0.51)	< 0.001
*P* for trend	< 0.001		< 0.001		< 0.001	

*eGDR*, estimated glucose disposal rate; *CKM*, cardiovascular-kidney-metabolic; *OR*, odds ratio; *CI*, confidence interval

### Subgroup and interaction analyses

**Fig. 4:**
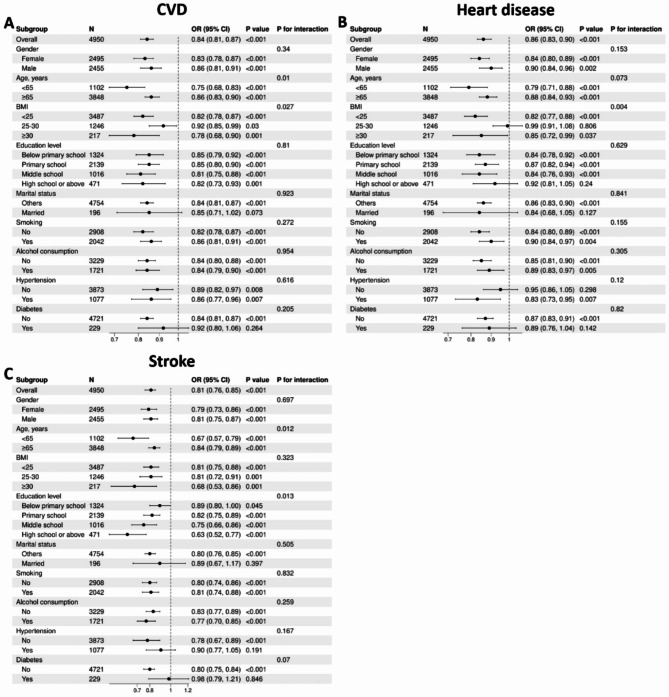
Subgroup analysis of the association between estimated glucose disposal rate and **A** CVD, **B** heart disease, and **C** stroke. *CVD*, cardiovascular disease; *OR*, odds ratio; *BMI*, body mass index

Subgroup and interaction analyses were performed by stratifying the population according to gender, age, BMI, education level, marital status, smoking status, alcohol consumption, hypertension, and diabetes. The relationship between eGDR and the incidence of CVD, heart disease, and stroke was consistent with the main results across most subgroups (Fig. 4).

Interaction effect analyses revealed that the association between eGDR and CVD and stroke was stronger (*P* for interaction < 0.05) in younger individuals (< 65 years) compared to older individuals (≥ 65 years). Additionally, BMI had a significant modifying effect on the relationship between eGDR and both CVD and heart disease (*P* for interaction < 0.05). The relationship between eGDR and stroke incidence was also significantly modified by education level (*P* for interaction = 0.013).

### Feature selection in machine learning model

**Fig. 5:**
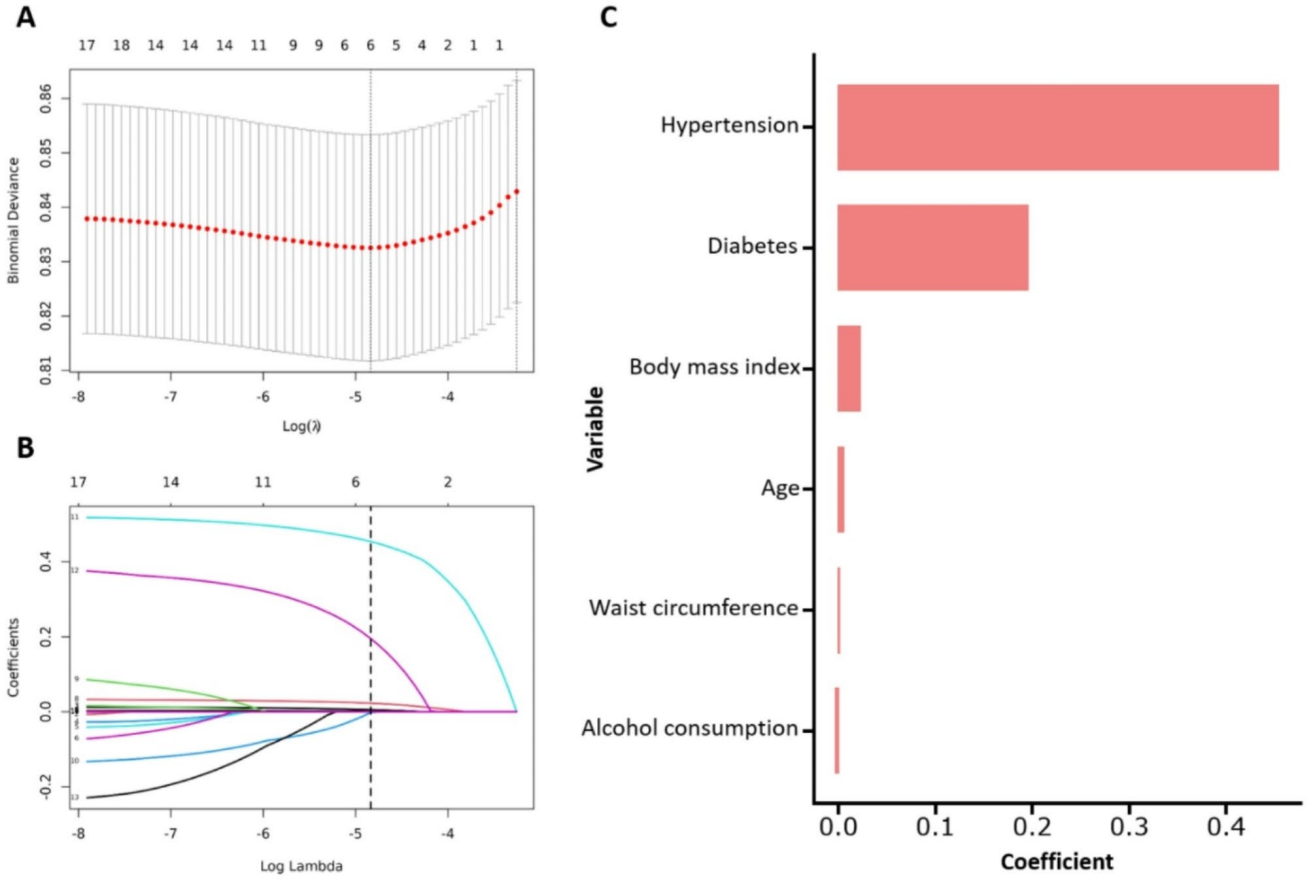
Feature selection based on the LASSO algorithm. **A** Selection of the tuning parameter (λ) in the LASSO model via 10-fold cross-validation based on minimum criteria. The optimal λ value of 0.008. **B** The LASSO coefficient profiles of clinical features. **C** The coefficients of LASSO regression analysis. *LASSO*, least absolute shrinkage and selection operator

Feature selection was performed using the LASSO algorithm (Fig. 5), which identified six key variables as significant predictors of adverse outcomes: hypertension, diabetes, age, BMI, WC, and alcohol consumption status. The correlation matrix for the study variables is shown in Supplementary Fig. 3, with significant relationships highlighted. Supplementary Fig. 4 displays the distributions of these variables used to develop the basic predictive model for CVD, heart disease, and stroke.

### Model development and validation

The dataset was randomly divided into training and testing sets in a 7:3 ratio for model development and validation. The comparisons of baseline characteristics are provided in Supplementary Table 4. The performance of the seven basic machine learning models for CVD, heart disease, and stroke is detailed in Supplementary Table 5. In the CVD basic model, KNN demonstrated the highest AUC, with a value of 0.840, followed by AdaBoost (AUC = 0.755), XGBoost (AUC = 0.753), SVM (AUC = 0.742), RF (AUC = 0.729), GNB (AUC = 0.727), and LightGBM (AUC = 0.617). Similarly, in the heart disease and stroke basic models, KNN outperformed the other machine learning models based on their concordance statistics (AUC). Therefore, the KNN algorithm was selected to further evaluate the performance of the modified machine learning model, which incorporated the eGDR index.

### Incremental predictive value of the eGDR index

**Fig. 6:**
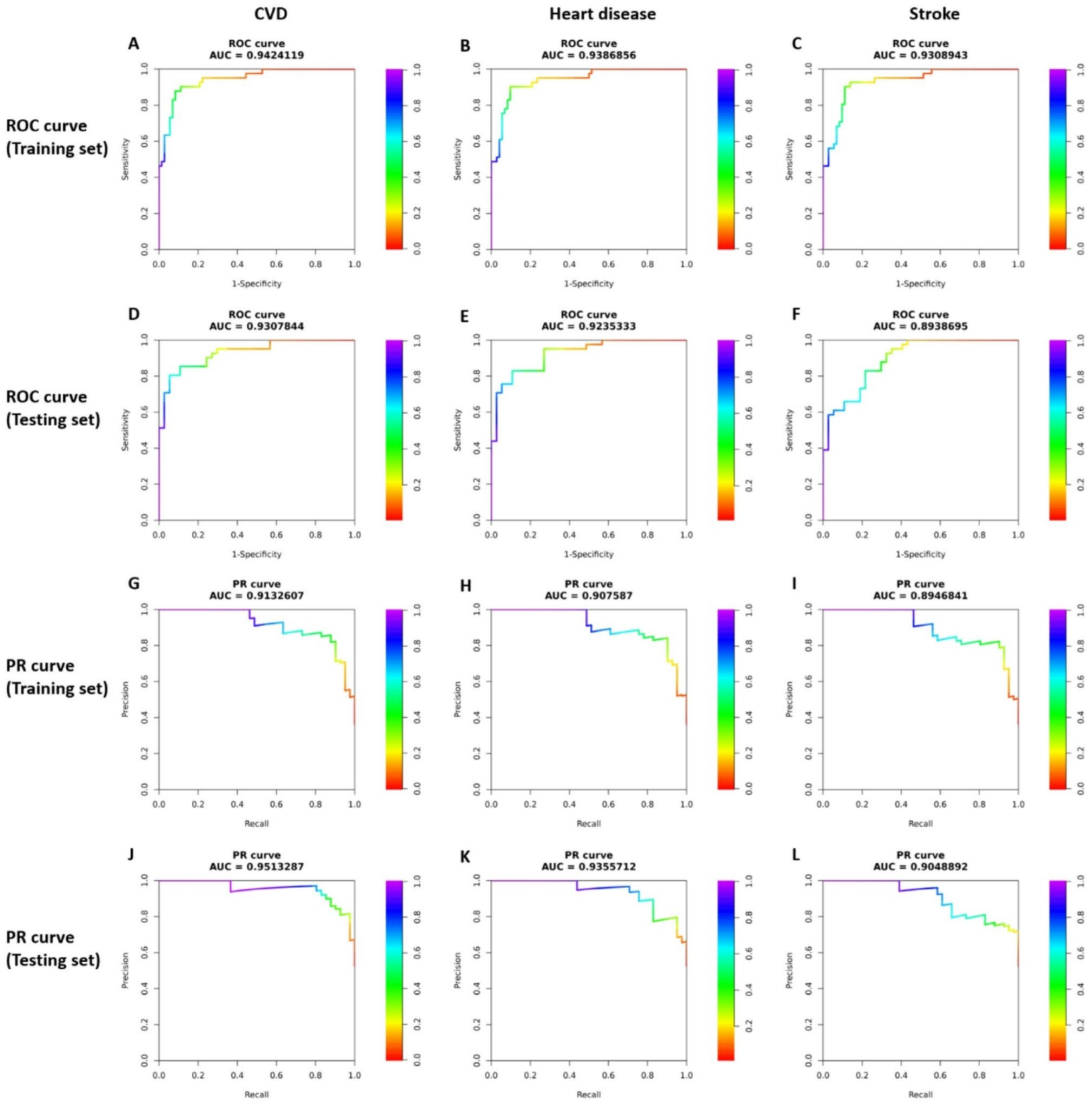
ROC and PR curves of the modified ML model, which incorporated the estimated glucose disposal rate, were plotted for predicting CVD, heart disease, and stroke in both the training and testing sets. **A–F** ROC curves of the modified ML model for predicting CVD, heart disease, and stroke in both the training and testing sets. **G–L** PR curves of the modified ML model for predicting CVD, heart disease, and stroke in both the training and testing sets. *ROC*, receiver operating characteristic; *PR*, precision-recall; *ML*, machine learning; *CVD*, cardiovascular disease; *AUC*, area under the curve

The incremental predictive value of the eGDR index for CVD, heart disease, and stroke was assessed using ROC and precision-recall curves, as shown in Fig. 6. The addition of the index to the basic model improved the AUC. Notably, in the modified CVD model, the AUC reached 0.942 and 0.931 in the training and testing sets, respectively. The AUPRC also showed good performance, with values of 0.913 and 0.951 in the training and testing sets, respectively. Similarly, in the heart disease and stroke models, the AUC and AUPRC demonstrated perfect predictive value.

DCA indicated that the modified machine learning model provided a superior net benefit across a range of threshold probabilities in both the training and testing sets, underscoring its clinical utility for decision-making (Supplementary Fig. 5, Panels A-F). Calibration curves showed that the model demonstrated strong calibration in both the training and testing sets. The Hosmer-Lemeshow test further supported the good model fit, with *P* values greater than 0.05, indicating no significant deviation between predicted and observed outcomes (Supplementary Fig. 5, Panels G-L). Overall, integrating the eGDR index enhanced risk stratification and discrimination for adverse cardiovascular outcomes in individuals with CKM syndrome.

## Discussion

Based on a comprehensive literature review, this study is the first to compare the predictive value of the eGDR index with six commonly used IR indices (TyG, TyG-WC, TyG-BMI, TyG-WHtR, TG/HDL-C, and METS-IR) for CVD events within the context of CKM syndrome. More importantly, we further assessed the incremental predictive value of the eGDR index for CVD, heart disease, and stroke using machine learning and multidimensional approaches. The key findings of our study are as follows: (1) The eGDR index was inversely associated with the risk of CVD events in individuals with CKM syndrome, a relationship that remained consistent across various factors such as age, gender, BMI, hypertension status, diabetes status, and others; (2) Participants in the highest quartile category of eGDR had adjusted ORs of 0.48 (95% CI 0.38–0.61) for CVD, 0.51 (95% CI 0.36–0.73) for heart disease, and 0.34 (95% CI 0.25–0.51) for stroke, compared to those in the lowest quartile; (3) The eGDR index outperformed six other IR indices in predicting CVD, heart disease, and stroke at the population level; and (4) Incorporating the eGDR index into predictive models significantly improved prediction performance for CVD events, yielding promising results. In summary, our study enhances risk stratification and may support the early identification of high-risk individuals within this population.

CKM syndrome is a significant global public health concern. The AHA emphasizes the urgent need for early screening in individuals at stages 0 to 3 of CKM syndrome, especially to prevent CVD events [1]. Reliable surrogate markers of IR provide valuable insights into the relationship between metabolic dysfunction and adverse CVD outcomes. In particular, IR indices have been increasingly recognized as independent risk factors for CVD events, even in individuals with CKM syndrome [7, 25–27]. Liao et al. [10] demonstrated a negative linear relationship between the eGDR index and CVD in diabetic or prediabetic populations, showing that it has significantly higher predictive value than other IR surrogates. Moreover, even among individuals without diabetes, eGDR has been associated with an increased risk of CVD events and long-term mortality [15, 20]. Similarly, Huang et al. [14] found that eGDR was inversely associated with the incidence of various CVD events, including myocardial infarction, heart failure, atrial fibrillation, and ischemic stroke in the general population. Notably, it also outperformed TyG, TyG-WC, TyG-BMI, TyG-WHtR, TG/HDL-C, and METS-IR in predicting these outcomes in clinical practice. Furthermore, eGDR is strongly associated with metabolic syndrome and shows superior predictive value for all-cause mortality compared to other IR indices, such as TyG [28]. However, in the context of CKM syndrome, Tian et al. [25] investigated the association between eGDR and CVDs but did not consider other IR indices or compare their predictive value for CVD events, leaving a gap in the current understanding of their relative efficacy.

In our study, we further compared the performance of eGDR with six other commonly used IR indices, including TyG, TyG-WC, TyG-BMI, TyG-WHtR, TG/HDL-C, and METS-IR, for predicting the incidence of CVD events. Consistent with previous findings, we found that the eGDR index demonstrated superior predictive value compared to the other commonly used IR indices, further underscoring its potential as an effective tool in clinical risk assessment.

Based on a large-scale cohort, our study prospectively investigated the relationship between eGDR and CVD events, including heart disease and stroke, in individuals with CKM syndrome. After fully adjusting for covariates, we observed a significantly inverse linear relationship between eGDR and the incidence of all three outcomes. Participants in the highest eGDR level (> 11.31) had a 52% lower risk of CVD (OR: 0.48, 95% CI:0.38–0.61), a 49% lower risk of heart disease (OR: 0.51, 95% CI 0.36–0.73), and a 66% lower risk of stroke (OR: 0.34, 95% CI 0.25–0.51) compared to those in the lowest level. Furthermore, each 1-unit increase in eGDR was associated with a 14%, 14%, and 19% lower risk of CVD, heart disease, and stroke, respectively. These findings are consistent with those of Huang et al. [14], who reported that each 1-unit increase in eGDR was associated with a 12%, 20%, 15%, and 13% lower risk of myocardial infarction, heart failure, atrial fibrillation, and ischemic stroke, respectively, in the general population. Similarly, Zhang et al. [20] found that a 1 standard deviation increase in eGDR was associated with a 17% lower risk for CVD, a 13% decreased risk for heart disease, and a 30% lower risk for stroke in individuals without diabetes. Additionally, Yi et al. [29] demonstrated that each 1 standard deviation increase in eGDR was linked to a 17% lower risk for atherosclerotic CVD in the general population. These findings reinforce the growing body of epidemiological evidence supporting the eGDR index as a robust and reliable risk stratification tool for cardiovascular and cerebrovascular events.

In addition to the overall results, we performed subgroup and interaction analyses, stratifying the population based on gender, age, BMI, education level, marital status, smoking status, alcohol consumption, hypertension, and diabetes. These analyses underscore the utility of eGDR in risk stratification across various demographic and clinical factors, further enhancing its relevance and applicability in populations affected by CKM syndrome.

Recent advances in machine learning within healthcare have significantly enhanced disease risk prediction and personalized treatment [30]. Machine learning techniques excel at identifying patterns and classifications in medical data, surpassing traditional statistical methods, and have been successfully implemented to improve patient care [17, 18, 30]. In this study, using machine learning models, we identified six key variables as significant predictors of adverse outcomes: hypertension, diabetes, age, BMI, WC, and alcohol consumption status. These clinical parameters, readily available in everyday clinical practice, offer valuable insights for predicting and preventing CVD events in this population. Hypertension and diabetes are prevalent chronic diseases and well-established risk factors for CVD [31, 32]. These conditions play a significant role in the progression of cardiovascular events, especially in individuals with CKM syndrome [1]. For instance, T2D is associated with a two- to four-fold increased risk of CVD, while CKD affects nearly 40% of individuals with T2D [4]. The pathophysiology of hypertension and diabetes is closely linked to metabolic abnormalities, with IR playing a pivotal role [1]. Age is a non-modifiable risk factor strongly associated with the development of CVD events [33]. As individuals age, the risk of atherosclerosis, coronary artery disease, and other cardiovascular conditions increases [33]. In our study, individuals with CVD and stroke were significantly older than those without these events. Elevated BMI and WC, which indicate obesity and visceral fat, exacerbate IR, inflammation, and arterial stiffness, further increasing the risk for cardiovascular events [25]. As reported in a recent study, BMI partially mediated the association between eGDR and the risk of CVD events [25]. Excessive alcohol consumption also elevates risk by negatively impacting blood pressure, heart function, and metabolic health [34, 35]. Of course, this association is complex and sometimes contradictory. Collectively, these factors underscore the multifaceted nature of cardiovascular risk in CKM syndrome.

In our study, the KNN model outperformed the other models, demonstrating superior predictive accuracy. This work presents a novel application of machine learning in assessing CKM syndrome. More importantly, incorporating eGDR into a machine learning framework enables the early identification of individuals at the highest risk for CVD events, allowing for timely and targeted interventions. This approach aligns with the principles of precision medicine, enabling clinicians to achieve more precise risk stratification and tailor interventions for high-risk patients, with the potential to optimize resource allocation and improve patient outcomes [31, 36].

Our study has several strengths. First, it is the first to employ machine learning and multidimensional approaches to investigate the incremental predictive performance of the eGDR index for CVD events in the context of CKM syndrome. Additionally, we compared eGDR with six commonly used IR indices, including TyG, TyG-WC, TyG-BMI, TyG-WHtR, TG/HDL-C, and METS-IR. Second, we utilized data from a large-scale national longitudinal survey. The large sample size and long-term follow-up provided a robust dataset, ensuring high statistical power and the reliability of the results. Furthermore, we adjusted for multiple confounding factors, allowing for a more accurate understanding of the associations between eGDR and CVD events in individuals with CKM syndrome. Subgroup analyses were conducted to further ensure the reliability and robustness of our findings. Finally, we employed ROC and precision-recall curves, calibration curves, and DCA analyses to thoroughly assess the performance of our models.

Despite the strengths of our study, several limitations must be acknowledged. First, as with other studies, the use of self-reported CVD outcomes may introduce bias. However, the CHARLS study implemented rigorous quality control measures, including face-to-face interviews, structured questionnaires, and validation of CVD history by a review committee, to ensure data accuracy. Second, the lack of time-to-event analysis is another limitation. We were unable to assess the impact of time on the relationship between eGDR and CVD risk. Future studies should incorporate time-to-event analysis to provide a more comprehensive evaluation of its effects. Third, while our machine learning models demonstrated excellent predictive performance, external validation in independent cohorts is needed to confirm the generalizability of our findings. Fourth, the study population was limited to individuals from China, and further research is needed to determine whether these results are applicable to other ethnic groups. Finally, although our model was adjusted for covariates, it could not eliminate the effect of unmeasured confounders. Future studies should incorporate additional biomarkers and clinical variables to assess the incremental predictive value of eGDR for CVD events more comprehensively. Despite these limitations, the innovative approach and reliability of this study provide valuable insights for future research in this field.

## Conclusion

In conclusion, our study highlights the superior predictive value of eGDR for CVD events in individuals with CKM syndrome stages 0–3, particularly when compared to other IR indices. Individuals with lower eGDR levels were found to be at a higher risk for future CVD events. Incorporating eGDR into machine learning models significantly enhances risk stratification, offering a promising tool for the early identification of high-risk individuals and enabling timely, targeted interventions. Future research should aim to validate these findings across diverse populations.

## Electronic supplementary material

Below is the link to the electronic supplementary material.

Supplementary Material 1.

## Data Availability

The data supporting the findings of this study are available the CHARLS website (http://charls.pku.edu.cn/en).

## Abbreviations

AHA: American Heart Association
CKM: Cardiovascular-kidney-metabolic
CKD: Chronic kidney disease
CVD: Cardiovascular diseases
HF: Heart failure
T2D: Type 2 diabetes
IR: Insulin resistance
eGDR: Estimated glucose disposal rate
WC: Waist circumference
HbA1c: Glycosylated hemoglobin A1c
TyG: Triglyceride–glucose
TyG-WC: TyG-waist circumference
TyG-BMI: TyG-body mass index
TyG-WHtR: TyG-waist-to-height ratio
TG/HDL-C: Triglyceride-to-high density lipoprotein cholesterol ratio
METS-IR: Metabolic score for insulin resistance
CHARLS: China Health and Retirement Longitudinal Study
SBP: Systolic blood pressure
DBP: Diastolic blood pressure
TC: Total cholesterol
LDL-C: Low density lipoprotein cholesterol
Scr: Serum creatinine
FBG: Fasting blood glucose
UA: Uric acid
LASSO: Least absolute shrinkage and selection operator
AdaBoost: Adaptive Boosting
KNN: K-Nearest Neighbor
LightGBM: Light Gradient Boosting Machine
RF: Random Forest
SVM: Support Vector Machine
XGBoost: EXtreme Gradient Boosting
GNB: Gaussian Naive Bayes
ANOVA: Analysis of variance
VIF: Variance inflation factor
RCS: Restricted cubic splines
AIC: Akaike information criterion
ROC: Receiver operating characteristic
AUC: Area under the ROC curve
AUPRC: Area under the precision-recall curve
DCA: Decision curve analysis
OR: Odds ratio

## References

[1] Ndumele CE, Rangaswami J, Chow SL, et al. Cardiovascular-kidney-metabolic health: a presidential advisory from the American heart association. Circulation. 2023;148(20):1606–35. 37807924 10.1161/CIR.0000000000001184

[2] Marassi M, Fadini GP. The cardio-renal-metabolic connection: a review of the evidence. Cardiovasc Diabetol. 2023;22(1):195. 37525273 10.1186/s12933-023-01937-x PMC10391899

[3] Maack C, Lehrke M, Backs J, et al. Heart failure and diabetes: metabolic alterations and therapeutic interventions: a state-of-the-art review from the translational research committee of the heart failure Association-European society of cardiology. Eur Heart J. 2018;39(48):4243–54. 30295797 10.1093/eurheartj/ehy596 PMC6302261

[4] Seferović PM, Petrie MC, Filippatos GS, et al. Type 2 diabetes mellitus and heart failure: a position statement from the heart failure association of the European society of cardiology. Eur J Heart Fail. 2018;20(5):853–72. 29520964 10.1002/ejhf.1170

[5] Damman K, Valente MA, Voors AA, et al. Renal impairment, worsening renal function, and outcome in patients with heart failure: an updated meta-analysis. Eur Heart J. 2014;35(7):455–69. 24164864 10.1093/eurheartj/eht386

[6] Ostrominski JW, Arnold SV, Butler J, et al. Prevalence and overlap of cardiac, renal, and metabolic conditions in US adults, 1999–2020. JAMA Cardiol. 2023;8(11):1050–60. 37755728 10.1001/jamacardio.2023.3241 PMC10535010

[7] Li W, Shen C, Kong W, et al. Association between the triglyceride glucose-body mass index and future cardiovascular disease risk in a population with cardiovascular-kidney-metabolic syndrome stage 0–3: a nationwide prospective cohort study. Cardiovasc Diabetol. 2024;23(1):292. 39113004 10.1186/s12933-024-02352-6 PMC11308445

[8] Malik S, Wong ND, Franklin SS, et al. Impact of the metabolic syndrome on mortality from coronary heart disease, cardiovascular disease, and all causes in United States adults. Circulation. 2004;110(10):1245–50. 15326067 10.1161/01.CIR.0000140677.20606.0E

[9] Zabala A, Darsalia V, Lind M, et al. Estimated glucose disposal rate and risk of stroke and mortality in type 2 diabetes: a nationwide cohort study. Cardiovasc Diabetol. 2021;20(1):202. 34615525 10.1186/s12933-021-01394-4 PMC8495918

[10] Liao J, Wang L, Duan L, et al. Association between estimated glucose disposal rate and cardiovascular diseases in patients with diabetes or prediabetes: a cross-sectional study. Cardiovasc Diabetol. 2025;24(1):13. 39806389 10.1186/s12933-024-02570-y PMC11730478

[11] Yan L, Zhou Z, Wu X, et al. Association between the changes in the estimated glucose disposal rate and new-onset cardiovascular disease in middle-aged and elderly individuals: a nationwide prospective cohort study in China. Diabetes Obes Metab. 2025;27(4):1859–67. 39762991 10.1111/dom.16179 PMC11885094

[12] Guo R, Tong J, Cao Y, et al. Association between estimated glucose disposal rate and cardiovascular mortality across the spectrum of glucose tolerance in the US population. Diabetes Obes Metab. 2024;26(12):5827–35. 39295089 10.1111/dom.15954

[13] Ichikawa T, Hashimoto Y, Okamura T et al. Estimated glucose disposal rate predicts the risk of incident metabolic dysfunction-associated steatotic liver disease. Endocr Pract. 2025:S1530–X891(25)00020– 5. 10.1016/j.eprac.2025.01.002 39818319

[14] Huang H, Xiong Y, Zhou J, et al. The predictive value of estimated glucose disposal rate and its association with myocardial infarction, heart failure, atrial fibrillation and ischemic stroke. Diabetes Obes Metab. 2025;27(3):1359–68. 39743837 10.1111/dom.16132

[15] He HM, Xie YY, Chen Q, et al. The additive effect of the triglyceride–glucose index and estimated glucose disposal rate on long-term mortality among individuals with and without diabetes: a population-based study. Cardiovasc Diabetol. 2024;23(1):307. 39175051 10.1186/s12933-024-02396-8 PMC11342524

[16] Jiang L, Zhu T, Song W, et al. Assessment of six insulin resistance surrogate indexes for predicting stroke incidence in Chinese middle-aged and elderly populations with abnormal glucose metabolism: a nationwide prospective cohort study. Cardiovasc Diabetol. 2025;24(1):56. 39915878 10.1186/s12933-025-02618-7 PMC11804005

[17] Huang Q, Zou X, Lian Z, et al. Predicting cardiovascular outcomes in Chinese patients with type 2 diabetes by combining risk factor trajectories and machine learning algorithm: a cohort study. Cardiovasc Diabetol. 2025;24(1):61. 39920715 10.1186/s12933-025-02611-0 PMC11806858

[18] Oikonomou EK, Khera R. Machine learning in precision diabetes care and cardiovascular risk prediction. Cardiovasc Diabetol. 2023;22(1):259. 37749579 10.1186/s12933-023-01985-3 PMC10521578

[19] Zhao Y, Hu Y, Smith JP, et al. Cohort profile: the China health and retirement longitudinal study (CHARLS). Int J Epidemiol. 2014;43(1):61–8. 23243115 10.1093/ije/dys203 PMC3937970

[20] Zhang Z, Zhao L, Lu Y, et al. Insulin resistance assessed by estimated glucose disposal rate and risk of incident cardiovascular diseases among individuals without diabetes: findings from a nationwide, population based, prospective cohort study. Cardiovasc Diabetol. 2024;23(1):194. 38844981 10.1186/s12933-024-02256-5 PMC11157942

[21] Li H, Zheng D, Li Z, et al. Association of depressive symptoms with incident cardiovascular diseases in middle-aged and older Chinese adults. JAMA Netw Open. 2019;2(12):e1916591. 31800066 10.1001/jamanetworkopen.2019.16591 PMC6902756

[22] Whelton PK, Carey RM, Aronow WS, et al. 2017 ACC/AHA/AAPA/ABC/ACPM/AGS/APhA/ASH/ASPC/NMA/PCNA guideline for the prevention, detection, evaluation, and management of high blood pressure in adults: a report of the American college of cardiology/american heart association task force on clinical practice guidelines. Hypertension. 2018;71(6):e13–115. 29133356 10.1161/HYP.0000000000000065

[23] American Diabetes Association Professional Practice Committee. 2. Diagnosis and classification of diabetes: standards of care in Diabetes-2024. Diabetes Care. 2024;47(Suppl 1):S20–42. 38078589 10.2337/dc24-S002 PMC10725812

[24] Lin J, Chen Y, Xu M, et al. Association and predictive ability between significant perioperative cardiovascular adverse events and stress glucose rise in patients undergoing non-cardiac surgery. Cardiovasc Diabetol. 2024;23(1):445. 39695608 10.1186/s12933-024-02542-2 PMC11657823

[25] Tian J, Chen H, Luo Y, et al. Association between estimated glucose disposal rate and prediction of cardiovascular disease risk among individuals with cardiovascular-kidney-metabolic syndrome stage 0–3: a nationwide prospective cohort study. Diabetol Metab Syndr. 2025;17(1):58. 39953554 10.1186/s13098-025-01626-7 PMC11827371

[26] Hu Y, Liang Y, Li J, et al. Correlation between atherogenic index of plasma and cardiovascular disease risk across Cardiovascular-kidney-metabolic syndrome stages 0–3: a nationwide prospective cohort study. Cardiovasc Diabetol. 2025;24(1):40. 39856691 10.1186/s12933-025-02593-z PMC11763136

[27] Zheng G, Jin J, Wang F, et al. Association between atherogenic index of plasma and future risk of cardiovascular disease in individuals with cardiovascular-kidney-metabolic syndrome stages 0–3: a nationwide prospective cohort study. Cardiovasc Diabetol. 2025;24(1):22. 39827127 10.1186/s12933-025-02589-9 PMC11743013

[28] Chen X, Li A, Ma Q. Association of estimated glucose disposal rate with metabolic syndrome prevalence and mortality risks: a population-based study. Cardiovasc Diabetol. 2025;24(1):38. 39844166 10.1186/s12933-025-02599-7 PMC11756087

[29] Yi J, Qu C, Li X, et al. Insulin resistance assessed by estimated glucose disposal rate and risk of atherosclerotic cardiovascular diseases incidence: the multi-ethnic study of atherosclerosis. Cardiovasc Diabetol. 2024;23(1):349. 39342205 10.1186/s12933-024-02437-2 PMC11439291

[30] Ngiam KY, Khor IW. Big data and machine learning algorithms for health-care delivery. Lancet Oncol. 2019;20(5):e262–73. 31044724 10.1016/S1470-2045(19)30149-4

[31] Li C, Zhang Z, Luo X, et al. The triglyceride–glucose index and its obesity-related derivatives as predictors of all-cause and cardiovascular mortality in hypertensive patients: insights from NHANES data with machine learning analysis. Cardiovasc Diabetol. 2025;24(1):47. 39881352 10.1186/s12933-025-02591-1 PMC11780913

[32] The Lancet Digital Health. Equitable precision medicine for type 2 diabetes. Lancet Digit Health. 2022;4(12):e850. 36427947 10.1016/S2589-7500(22)00217-5

[33] North BJ, Sinclair DA. The intersection between aging and cardiovascular disease. Circ Res. 2012;110(8):1097–108. 22499900 10.1161/CIRCRESAHA.111.246876 PMC3366686

[34] Hu C, Huang C, Li J, et al. Causal associations of alcohol consumption with cardiovascular diseases and all-cause mortality among Chinese males. Am J Clin Nutr. 2022;116(3):771–9. 35687413 10.1093/ajcn/nqac159

[35] Roerecke M. Alcohol’s impact on the cardiovascular system. Nutrients. 2021;13(10):3419. 34684419 10.3390/nu13103419 PMC8540436

[36] Nabrdalik K, Kwiendacz H, Irlik K, et al. Machine learning identification of risk factors for heart failure in patients with diabetes mellitus with metabolic dysfunction associated steatotic liver disease (MASLD): the silesia diabetes-heart project. Cardiovasc Diabetol. 2023;22(1):318. 37985994 10.1186/s12933-023-02014-z PMC10661663

