# Supplementary material for: Estimated glucose disposal rate outperforms other insulin resistance surrogates in predicting incident cardiovascular diseases in cardiovascular-kidney-metabolic syndrome stages 0–3 and the development of a machine learning prediction model: a nationwide prospective cohort study
Source: Cardiovasc Diabetol. 2025 Apr 16;24:163. doi: 10.1186/s12933-025-02729-1 (PMC12004813; doi:10.1186/s12933-025-02729-1)

**Supplementary Materials and Methods**

**Title:**

Estimated glucose disposal rate outperforms other insulin resistance surrogates in predicting incident cardiovascular diseases in cardiovascular-kidney-metabolic syndrome stages 0–3 and the development of a machine learning prediction model: a nationwide prospective cohort study

**Authors:**

Bingtian Dong^1^†, Yuping Chen^2,3^†, Xiaocen Yang^4^†, Zhengdong Chen^5,6^, Hua Zhang^7^, Yuan Gao^1^, Enfa Zhao^1^* and Chaoxue Zhang^1^*

†Bingtian Dong, Yuping Chen, and Xiaocen Yang contributed equally to this work.

***Correspondence:**

Chaoxue Zhang

E-mail: zcxay@163.com

OR

Enfa Zhao

E-mail: zhaoenfasy@163.com

**Supplementary Materials and Methods, Part I.** Definition of insulin resistance (IR) surrogate indices

In accordance with previous studies [1,2], the estimated glucose disposal rate (eGDR) index and other IR indices were calculated as follows:

1. eGDR = 21.158 − (0.09 × waist circumference [WC, cm]) − (3.407 × hyperetnsion [yes = 1/no = 0]) − (0.551 × glycosylated hemoglobin A1c [HbA1c, %]);
2. Triglyceride-glucose (TyG) index = Ln (fasting triglyceride [mg/dL] × fasting glucose [mg/dL]/2);
3. TyG-WC index = TyG index × WC (cm);
4. TyG-body mass index (TyG-BMI) index = TyG index × BMI;
5. TyG-waist-to-height ratio (TyG-WHtR) index = TyG index × WC (cm)/height (cm);
6. Triglyceride-to-high density lipoprotein cholesterol (HDL-C) ratio (TG/HDL-C) = fasting triglyceride (mg/dL)/fasting HDL-C (mg/dL).
7. Metabolic score for IR (METS-IR) = Ln (2 × fasting glucose [mg/dL] + fasting triglyceride [mg/dL]) × BMI/Ln (fasting HDL-C [mg/dL]).

**Supplementary Materials and Methods, Part II.** Overview of machine learning algorithms

**(1) Adaptive Boosting (AdaBoost):** AdaBoost is an ensemble learning method that combines multiple weak classifiers to create a strong classifier [3,4]. It works by assigning higher weights to misclassified instances in each iteration, improving the performance of the model incrementally. It's particularly useful for classification tasks.

**(2) K-Nearest Neighbor (KNN):** KNN is a simple, instance-based learning algorithm that classifies a data point based on the majority label of its nearest neighbors. The algorithm calculates the distance between the test point and all other points in the dataset and uses the labels of the k-nearest neighbors to predict the label of the test point [5]. It is widely used for classification and regression tasks.

**(3) Light Gradient Boosting Machine (LightGBM):** LightGBM is a gradient boosting framework that uses decision trees to build a strong predictive model. It is known for its speed and efficiency, especially with large datasets [6]. LightGBM uses a technique called histogram-based decision tree learning, which helps to speed up training while maintaining high accuracy.

**(4) Random Forest (RF):** RF is an ensemble learning method that constructs multiple decision trees during training and outputs the class that is the majority vote or the mean prediction of the individual trees [7]. It helps to reduce overfitting and improve model accuracy by averaging predictions from multiple trees.

**(5) Support Vector Machine (SVM):** SVM is a powerful classification algorithm that works by finding a hyperplane that best separates the classes in the feature space [8]. It tries to maximize the margin between the classes, which helps to improve the generalization ability of the model. SVM can be used for both linear and non-linear classification tasks using kernel functions.

**(6) eXtreme Gradient Boosting (XGBoost):** XGBoost is an optimized gradient boosting algorithm designed for speed and performance [9]. It builds trees sequentially and corrects errors made by previous trees. XGBoost is known for its regularization techniques, which help reduce overfitting and improve prediction accuracy, making it popular in machine learning competitions.

**(7) Gaussian Naive Bayes (GNB):** GNB is a probabilistic classification algorithm based on Bayes’ Theorem, assuming that the features follow a Gaussian (normal) distribution [10]. It calculates the probability of a class given the input features and classifies the data based on the highest posterior probability. It is simple, fast, and works well with categorical or continuous data.

**References**

1. Zabala A, Darsalia V, Lind M, et al. Estimated glucose disposal rate and risk of stroke and mortality in type 2 diabetes: a nationwide cohort study. Cardiovasc Diabetol. 2021;20(1):202.
2. He HM, Xie YY, Chen Q, et al. The additive effect of the triglyceride-glucose index and estimated glucose disposal rate on long-term mortality among individuals with and without diabetes: a population-based study. Cardiovasc Diabetol. 2024;23(1):307.
3. Freund Y, Schapire R. A decision-theoretic generalization of on-line learning and an application to boosting. J Comput Syst Sci. 1997;55(1):119-139.
4. Avidan S. Ensemble tracking. IEEE Trans Pattern Anal Mach Intell. 2007;29(2):261-271.
5. Rui F, Yeo YH, Xu L, et al. Development of a machine learning-based model to predict hepatic inflammation in chronic hepatitis B patients with concurrent hepatic steatosis: a cohort study. EClinicalMedicine. 2024;68:102419.
6. Talkhi N, Nooghabi MJ, Esmaily H, et al. Prediction of serum anti-HSP27 antibody titers changes using a light gradient boosting machine (LightGBM) technique. Sci Rep. 2023;13(1):12775.
7. Breiman L. Random forests. Mach Learn. 2001;45(1):5-32.
8. Fan R, Yu N, Li G, et al. Machine-learning model comprising five clinical indices and liver stiffness measurement can accurately identify MASLD-related liver fibrosis. Liver Int. 2024;44(3):749-759.
9. Ester M, Kriegel HP, Xu X. XGBoost: a scalable tree boosting system. In: Proceedings of the 22Nd ACM SIGKDD international conference on knowledge discovery and data mining (vol, pg 785, 2016). Geogr Anal; 2022.
10. Domingos P, Pazzani M. On the optimality of the simple Bayesian classifier under zero-one loss. Mach Learn. 1997;29(2-3):103-130.

**Supplementary Table 1.** Baseline characteristics of the study participants with and without CVD

| **Characteristics** | **Non-CVD**  **(n = 4,253)** | **CVD**  **(n = 697)** | ***P* value** |
| --- | --- | --- | --- |
| Age (years) | 73.28 ± 10.053 | 74.62 ± 9.056 | <0.001 |
| Female, n (%) | 2,129 (50.1%) | 366 (52.5%) | 0.230 |
| Education level, n (%) |  |  | 0.798 |
| Below primary school | 1,130 (26.6%) | 194 (27.8%) |  |
| Primary school | 1,835 (43.1%) | 304 (43.6%) |  |
| Middle school | 881 (20.7%) | 135 (19.4%) |  |
| High school or above | 407 (9.6%) | 64 (9.2%) |  |
| Marital status, n (%) |  |  | 0.615 |
| Married | 166 (3.9%) | 30 (4.3%) |  |
| Others | 4,087 (96.1%) | 667 (95.7%) |  |
| SBP (mmHg) | 129.49 ± 21.234 | 135.22 ± 22.526 | <0.001 |
| DBP (mmHg) | 75.17 ± 11.919 | 77.91 ± 12.848 | <0.001 |
| BMI (kg/m^2^) | 23.24 ± 3.809 | 24.12 ± 4.150 | <0.001 |
| WC (cm) | 83.61 ± 12.001 | 86.00 ± 13.486 | <0.001 |
| TG (mg/dL) | 126.51 ± 88.898 | 137.03 ± 98.763 | 0.008 |
| TC (mg/dL) | 193.16 ± 37.901 | 196.80 ± 37.241 | 0.017 |
| HDL-C (mg/dL) | 52.00 ± 15.343 | 50.95 ± 15.546 | 0.098 |
| LDL-C (mg/dL) | 116.54 ± 34.589 | 118.97 ± 35.426 | 0.093 |
| Scr (mg/dl) | 0.79 ± 0.277 | 0.78 ± 0.182 | 0.437 |
| FPG (mg/dL) | 108.87 ± 34.769 | 111.76 ± 35.317 | 0.045 |
| HbA1c (%) | 5.24 ± 0.768 | 5.31 ± 0.759 | 0.018 |
| UA, mg/dL | 4.47 ± 1.250 | 4.54 ± 1.311 | 0.219 |
| Smoking, n (%) | 1,750 (41.1%) | 292 (41.9%) | 0.711 |
| Alcohol consumption, n (%) | 1,494 (35.1%) | 227 (32.6%) | 0.188 |
| Hypertension, n (%) | 838 (19.7%) | 239 (34.3%) | <0.001 |
| Diabetes, n (%) | 179 (4.2%) | 50 (7.2%) | <0.001 |
| IR surrogate indices |  |  |  |
| eGDR | 10.08 ± 1.951 | 9.32 ± 2.301 | <0.001 |
| TyG | 8.65 ± 0.632 | 8.74 ± 0.671 | <0.001 |
| TyG-WC | 724.60 ± 128.523 | 754.19 ± 145.127 | <0.001 |
| TyG-BMI | 201.55 ± 39.973 | 211.78 ± 44.450 | <0.001 |
| TyG-WHtR | 4.60 ± 0.830 | 4.77 ± 0.924 | <0.001 |
| TG/HDL-C | 3.02 ± 4.020 | 3.38 ± 4.941 | 0.068 |
| METS-IR | 34.95 ± 8.212 | 36.82 ± 9.057 | <0.001 |

Abbreviations: *CVD*, cardiovascular disease; *eGDR*, estimated glucose disposal rate; *SBP*, systolic blood pressure; *DBP*, diastolic blood pressure; *BMI*, body mass index; *WC*, waist circumference; *TG*, triglyceride; *TC*, total cholesterol; *HDL-C*, high-density lipoprotein cholesterol; *LDL-C*, low-density lipoprotein cholesterol; *Scr*, serum creatinine; *FPG*, fasting plasma glucose; *HbA1c*, glycosylated hemoglobin A1c; *UA*, uric acid; *IR*, insulin resistance; *TyG*, triglyceride-glucose; *TyG-WC*, TyG-waist circumference; *TyG-BMI*, TyG-body mass index; *TyG-WHtR*, TyG-waist-to-height ratio; *TG/HDL-C*, triglyceride-to-high density lipoprotein cholesterol ratio; *METS-IR*, metabolic score for insulin resistance.

Data are presented as mean ± standard deviation or number (%)

**Supplementary Table 2.** Baseline characteristics of the study participants with and without heart disease

| **Characteristics** | **Non-heart disease**  **(n = 4,464)** | **Heart disease**  **(n = 486)** | ***P* value** |
| --- | --- | --- | --- |
| Age (years) | 73.39 ± 10.008 | 74.11 ± 9.148 | 0.105 |
| Female, n (%) | 2,216 (49.6%) | 279 (57.4%) | 0.001 |
| Education level, n (%) |  |  | 0.692 |
| Below primary school | 1,187 (26.6%) | 137 (28.2%) |  |
| Primary school | 1,937 (43.4%) | 202 (41.6%) |  |
| Middle school | 920 (20.6%) | 96 (19.8%) |  |
| High school or above | 420 (9.4%) | 51 (10.5%) |  |
| Marital status, n (%) |  |  | 0.952 |
| Married | 177 (4.0%) | 19 (3.9%) |  |
| Others | 4,287 (96.0%) | 467 (96.1%) |  |
| SBP (mmHg) | 129.99 ± 21.478 | 133.13 ± 21.623 | 0.002 |
| DBP (mmHg) | 75.38 ± 12.027 | 77.09 ± 12.563 | 0.004 |
| BMI (kg/m^2^) | 23.28 ± 3.832 | 24.13 ± 4.135 | <0.001 |
| WC (cm) | 83.77 ± 12.053 | 85.51 ± 13.819 | 0.008 |
| TG (mg/dL) | 127.62 ± 91.517 | 131.39 ± 79.592 | 0.329 |
| TC (mg/dL) | 193.41 ± 37.932 | 196.09 ± 36.791 | 0.129 |
| HDL-C (mg/dL) | 51.88 ± 15.367 | 51.65 ± 15.461 | 0.753 |
| LDL-C (mg/dL) | 116.71 ± 34.698 | 118.48 ± 34.869 | 0.287 |
| Scr (mg/dl) | 0.79 ± 0.273 | 0.77 ± 0.183 | 0.027 |
| FPG (mg/dL) | 109.07 ± 34.771 | 111.15 ± 35.631 | 0.222 |
| HbA1c (%) | 5.24 ± 0.765 | 5.33 ± 0.780 | 0.019 |
| UA, mg/dL | 4.48 ± 1.258 | 4.47 ± 1.273 | 0.922 |
| Smoking, n (%) | 1,852 (41.5%) | 190 (39.1%) | 0.309 |
| Alcohol consumption, n (%) | 1,564 (35.0%) | 157 (32.3%) | 0.230 |
| Hypertension, n (%) | 916 (20.5%) | 161 (33.1%) | <0.001 |
| Diabetes, n (%) | 192 (4.3%) | 37 (7.6%) | <0.001 |
| IR surrogate indices |  |  |  |
| eGDR | 10.04 ± 1.972 | 9.40 ± 2.347 | <0.001 |
| TyG | 8.65 ± 0.636 | 8.71 ± 0.663 | 0.082 |
| TyG-WC | 726.81 ± 129.570 | 746.74 ± 145.856 | 0.004 |
| TyG-BMI | 202.13 ± 40.297 | 210.97 ± 44.265 | <0.001 |
| TyG-WHtR | 4.61 ± 0.834 | 4.74 ± 0.940 | 0.003 |
| TG/HDL-C | 3.06 ± 4.154 | 3.16 ± 4.245 | 0.605 |
| METS-IR | 35.06 ± 8.273 | 36.61 ± 9.014 | <0.001 |

Abbreviations: *eGDR*, estimated glucose disposal rate; *SBP*, systolic blood pressure; *DBP*, diastolic blood pressure; *BMI*, body mass index; *WC*, waist circumference; *TG*, triglyceride; *TC*, total cholesterol; *HDL-C*, high-density lipoprotein cholesterol; *LDL-C*, low-density lipoprotein cholesterol; *Scr*, serum creatinine; *FPG*, fasting plasma glucose; *HbA1c*, glycosylated hemoglobin A1c; *UA*, uric acid; *IR*, insulin resistance; *TyG*, triglyceride-glucose; *TyG-WC*, TyG-waist circumference; *TyG-BMI*, TyG-body mass index; *TyG-WHtR*, TyG-waist-to-height ratio; *TG/HDL-C*, triglyceride-to-high density lipoprotein cholesterol ratio; *METS-IR*, metabolic score for insulin resistance.

Data are presented as mean ± standard deviation or number (%)

**Supplementary Table 3.** Baseline characteristics of the study participants with and without stroke

| **Characteristics** | **Non-stroke**  **(n = 4,687)** | **Stroke**  **(n = 263)** | ***P* value** |
| --- | --- | --- | --- |
| Age (years) | 73.34 ± 9.970 | 75.65 ± 8.900 | <0.001 |
| Female, n (%) | 2,380 (50.8%) | 115 (43.7%) | 0.026 |
| Education level, n (%) |  |  | 0.451 |
| Below primary school | 1,253 (26.7%) | 71 (27.0%) |  |
| Primary school | 2,015 (43.0%) | 124 (47.1%) |  |
| Middle school | 969 (20.7%) | 47 (17.9%) |  |
| High school or above | 450 (9.6%) | 21 (8.0%) |  |
| Marital status, n (%) |  |  | 0.849 |
| Married | 185 (3.9%) | 11 (4.2%) |  |
| Others | 4,502 (96.1%) | 252 (95.8%) |  |
| SBP (mmHg) | 129.76 ± 21.224 | 139.95 ± 24.187 | <0.001 |
| DBP (mmHg) | 75.31 ± 11.946 | 79.78 ± 13.784 | <0.001 |
| BMI (kg/m^2^) | 23.32 ± 3.851 | 24.21 ± 4.111 | <0.001 |
| WC (cm) | 83.75 ± 12.215 | 87.44 ± 12.329 | <0.001 |
| TG (mg/dL) | 126.90 ± 88.215 | 147.33 ± 121.821 | 0.008 |
| TC (mg/dL) | 193.39 ± 37.787 | 198.71 ± 38.235 | 0.029 |
| HDL-C (mg/dL) | 52.02 ± 15.364 | 48.95 ± 15.303 | 0.002 |
| LDL-C (mg/dL) | 116.64 ± 34.588 | 121.33 ± 36.697 | 0.044 |
| Scr (mg/dl) | 0.79 ± 0.270 | 0.82 ± 0.177 | 0.015 |
| FPG (mg/dL) | 109.03 ± 34.813 | 113.73 ± 35.421 | 0.037 |
| HbA1c (%) | 5.24 ± 0.769 | 5.29 ± 0.722 | 0.342 |
| UA, mg/dL | 4.47 ± 1.252 | 4.70 ± 1.359 | 0.008 |
| Smoking, n (%) | 1,917 (40.9%) | 125 (47.5%) | 0.034 |
| Alcohol consumption, n (%) | 1,629 (34.8%) | 92 (35.0%) | 0.940 |
| Hypertension, n (%) | 975 (20.8%) | 102 (38.8%) | <0.001 |
| Diabetes, n (%) | 211 (4.5%) | 18 (6.8%) | 0.078 |
| IR surrogate indices |  |  |  |
| eGDR | 10.03 ± 1.997 | 9.05 ± 2.208 | <0.001 |
| TyG | 8.65 ± 0.636 | 8.83 ± 0.661 | <0.001 |
| TyG-WC | 726.23 ± 130.621 | 773.95 ± 136.757 | <0.001 |
| TyG-BMI | 202.35 ± 40.525 | 214.44 ± 43.673 | <0.001 |
| TyG-WHtR | 4.61 ± 0.844 | 4.87 ± 0.844 | <0.001 |
| TG/HDL-C | 3.03 ± 4.054 | 3.79 ± 5.727 | 0.033 |
| METS-IR | 35.08 ± 8.305 | 37.52 ± 8.999 | <0.001 |

Abbreviations: *eGDR*, estimated glucose disposal rate; *SBP*, systolic blood pressure; *DBP*, diastolic blood pressure; *BMI*, body mass index; *WC*, waist circumference; *TG*, triglyceride; *TC*, total cholesterol; *HDL-C*, high-density lipoprotein cholesterol; *LDL-C*, low-density lipoprotein cholesterol; *Scr*, serum creatinine; *FPG*, fasting plasma glucose; *HbA1c*, glycosylated hemoglobin A1c; *UA*, uric acid; *IR*, insulin resistance; *TyG*, triglyceride-glucose; *TyG-WC*, TyG-waist circumference; *TyG-BMI*, TyG-body mass index; *TyG-WHtR*, TyG-waist-to-height ratio; *TG/HDL-C*, triglyceride-to-high density lipoprotein cholesterol ratio; *METS-IR*, metabolic score for insulin resistance.

Data are presented as mean ± standard deviation or number (%)

**Supplementary Table 4.** Baseline characteristics of the study participants in both the training and testing sets

| **Characteristics** | **Training set**  **(n = 3,466)** | **Testing set**  **(n = 1,484)** | ***P* value** |
| --- | --- | --- | --- |
| Age (years) | 73.65 ± 9.944 | 73.04 ± 9.883 | 0.047 |
| Female, n (%) | 1,742 (50.3%) | 753 (50.7%) | 0.756 |
| Education level, n (%) |  |  | 0.044 |
| Below primary school | 960 (27.7%) | 364 (24.5%) |  |
| Primary school | 1,486 (42.9%) | 653 (44.0%) |  |
| Middle school | 684 (19.7%) | 332 (22.4%) |  |
| High school or above | 336 (9.7%) | 135 (9.1%) |  |
| Marital status, n (%) |  |  | 0.661 |
| Married | 140 (4.0%) | 56 (3.8%) |  |
| Others | 3,326 (96.0%) | 1,428 (96.2%) |  |
| SBP (mmHg) | 130.58 ± 21.435 | 129.63 ± 21.679 | 0.158 |
| DBP (mmHg) | 75.66 ± 12.090 | 75.30 ± 12.092 | 0.344 |
| BMI (kg/m^2^) | 23.31 ± 3.837 | 23.50 ± 3.946 | 0.113 |
| WC (cm) | 83.75 ± 12.436 | 84.39 ± 11.786 | 0.087 |
| TG (mg/dL) | 127.27 ± 88.219 | 129.67 ± 95.359 | 0.406 |
| TC (mg/dL) | 193.63 ± 38.314 | 193.75 ± 36.676 | 0.920 |
| HDL-C (mg/dL) | 51.91 ± 15.401 | 51.73 ± 15.318 | 0.711 |
| LDL-C (mg/dL) | 116.83 ± 34.824 | 117.02 ± 34.471 | 0.855 |
| Scr (mg/dl) | 0.79 ± 0.274 | 0.78 ± 0.245 | 0.100 |
| FPG (mg/dL) | 108.89 ± 35.000 | 110.18 ± 34.518 | 0.228 |
| HbA1c (%) | 5.24 ± 0.766 | 5.25 ± 0.769 | 0.713 |
| UA, mg/dL | 4.49 ± 1.252 | 4.45 ± 1.275 | 0.279 |
| Smoking, n (%) | 1,436 (41.4%) | 606 (40.8%) | 0.697 |
| Alcohol consumption, n (%) | 1,185 (34.2%) | 536 (36.1%) | 0.192 |
| Hypertension, n (%) | 762 (22.0%) | 315 (21.2%) | 0.553 |
| Diabetes, n (%) | 160 (4.6%) | 69 (4.6%) | 0.959 |
| Heart disease, n (%) | 368 (10.6%) | 118 (8.0%) | 0.004 |
| Stroke, n (%) | 187 (5.4%) | 76 (5.1%) | 0.694 |
| CVD, n (%) | 517 (14.9%) | 180 (12.1%) | 0.010 |
| IR surrogate indices |  |  |  |
| eGDR | 9.99 ± 2.030 | 9.95 ± 2.000 | 0.546 |
| TyG | 8.65 ± 0.637 | 8.68 ± 0.643 | 0.212 |
| TyG-WC | 726.43 ± 132.038 | 734.23 ± 129.698 | 0.054 |
| TyG-BMI | 202.29 ± 40.252 | 204.64 ± 41.967 | 0.067 |
| TyG-WHtR | 4.61 ± 0.849 | 4.65 ± 0.838 | 0.082 |
| TG/HDL-C | 3.00 ± 3.511 | 3.21 ± 5.385 | 0.166 |
| METS-IR | 35.06 ± 8.088 | 35.56 ± 8.958 | 0.064 |

Abbreviations: *CVD*, cardiovascular disease; *eGDR*, estimated glucose disposal rate; *SBP*, systolic blood pressure; *DBP*, diastolic blood pressure; *BMI*, body mass index; *WC*, waist circumference; *TG*, triglyceride; *TC*, total cholesterol; *HDL-C*, high-density lipoprotein cholesterol; *LDL-C*, low-density lipoprotein cholesterol; *Scr*, serum creatinine; *FPG*, fasting plasma glucose; *HbA1c*, glycosylated hemoglobin A1c; *UA*, uric acid; *IR*, insulin resistance; *TyG*, triglyceride-glucose; *TyG-WC*, TyG-waist circumference; *TyG-BMI*, TyG-body mass index; *TyG-WHtR*, TyG-waist-to-height ratio; *TG/HDL-C*, triglyceride-to-high density lipoprotein cholesterol ratio; *METS-IR*, metabolic score for insulin resistance.

Data are presented as mean ± standard deviation or number (%)

**Supplementary Table 5.** The performance of seven basic machine learning models constructed with CVD, heart disease, and stroke as the outcome variables

| **Basic model** | **AUC** | **Sensitivity** | **Specificity** | **Accuracy** | **F1-score** |
| --- | --- | --- | --- | --- | --- |
| **CVD** |  |  |  |  |  |
| AdaBoost | 0.755 (0.723-0.786) | 0.706 (0.648-0.764) | 0.687 (0.623-0.751) | 0.689 (0.640-0.739) | 0.402 (0.373-0.431) |
| KNN | 0.840 (0.821-0.860) | 0.603 (0.535-0.671) | 1.000 (1.000-1.000) | 0.603 (0.535-0.671) | 0.426 (0.381-0.470) |
| LightGBM | 0.617 (0.590-0.645) | 0.485 (0.200-0.770) | 0.713 (0.529-0.898) | 0.680 (0.558-0.802) | 0.268 (0.160-0.375) |
| RF | 0.729 (0.698-0.760) | 0.756 (0.708-0.804) | 0.576 (0.528-0.624) | 0.602 (0.567-0.637) | 0.355 (0.349-0.362) |
| SVM | 0.742 (0.710-0.774) | 0.792 (0.759-0.824) | 0.553 (0.504-0.602) | 0.587 (0.550-0.625) | 0.357 (0.346-0.369) |
| XGBoost | 0.753 (0.708-0.797) | 0.696 (0.600-0.792) | 0.708 (0.654-0.761) | 0.706 (0.670-0.741) | 0.433 (0.398-0.469) |
| GNB | 0.727 (0.694-0.760) | 0.690 (0.638-0.743) | 0.581 (0.518-0.643) | 0.662 (0.637-0.687) | 0.371 (0.355-0.388) |
| **Heart disease** |  |  |  |  |  |
| AdaBoost | 0.846 (0.827-0.865) | 1.000 (1.000-1.000) | 0.551 (0.548-0.553) | 0.616 (0.613-0.618) | 0.429 (0.427-0.431) |
| KNN | 0.851 (0.834-0.869) | 1.000 (1.000-1.000) | 0.570 (0.522-0.618) | 0.632 (0.591-0.673) | 0.444 (0.417-0.470) |
| LightGBM | 0.708 (0.659-0.758) | 0.706 (0.656-0.755) | 0.616 (0.539-0.693) | 0.629 (0.570-0.688) | 0.355 (0.337-0.374) |
| RF | 0.737 (0.703-0.771) | 0.627 (0.474-0.779) | 0.733 (0.630-0.836) | 0.717 (0.647-0.788) | 0.389 (0.341-0.437) |
| SVM | 0.790 (0.739-0.840) | 0.547 (0.516-0.578) | 0.960 (0.959-0.961) | 0.900 (0.896-0.904) | 0.612 (0.591-0.633) |
| XGBoost | 0.800 (0.764-0.836) | 0.813 (0.787-0.839) | 0.637 (0.606-0.668) | 0.663 (0.632-0.693) | 0.411 (0.383-0.439) |
| GNB | 0.826 (0.794-0.859) | 1.000 (1.000-1.000) | 0.549 (0.505-0.593) | 0.610 (0.578-0.643) | 0.409 (0.401-0.418) |
| **Stroke** |  |  |  |  |  |
| AdaBoost | 0.830 (0.810-0.850) | 1.000 (1.000-1.000) | 0.493 (0.462-0.523) | 0.566 (0.540-0.592) | 0.221 (0.197-0.245) |
| KNN | 0.877 (0.844-0.911) | 0.821 (0.755-0.886) | 0.765 (0.688-0.842) | 0.773 (0.714-0.832) | 0.515 (0.465-0.565) |
| LightGBM | 0.855 (0.818-0.892) | 0.821 (0.779-0.863) | 0.724 (0.694-0.755) | 0.739 (0.718-0.760) | 0.487 (0.475-0.499) |
| RF | 0.813 (0.768-0.858) | 0.550 (0.547-0.553) | 0.961 (0.950-0.971) | 0.901 (0.893-0.910) | 0.618 (0.599-0.636) |
| SVM | 0.835 (0.810-0.859) | 1.000 (1.000-1.000) | 0.554 (0.454-0.654) | 0.619(0.533-0.704) | 0.433 (0.379-0.486) |
| XGBoost | 0.845 (0.826-0.864) | 1.000 (1.000-1.000) | 0.551 (0.538-0.564) | 0.616 (0.605-0.627) | 0.430 (0.422-0.437) |
| GNB | 0.847 (0.828-0.866) | 1.000 (1.000-1.000) | 0.551 (0.540-0.562) | 0.616 (0.606-0.626) | 0.430 (0.423-0.436) |

Abbreviations: *CVD*, cardiovascular disease; *AUC*, area under the receiver operating characteristic curve; *AdaBoost*, Adaptive Boosting; *KNN*, K-Nearest Neighbor; *LightGBM*, Light Gradient Boosting Machine; *RF*, Random Forest; *SVM*, Support Vector Machine; *XGBoost*, eXtreme Gradient Boosting; *GNB*, Gaussian Naive Bayes

**Supplementary Fig. 1** Distribution of the eGDR index. Abbreviations: *eGDR*, estimated glucose disposal rate

**
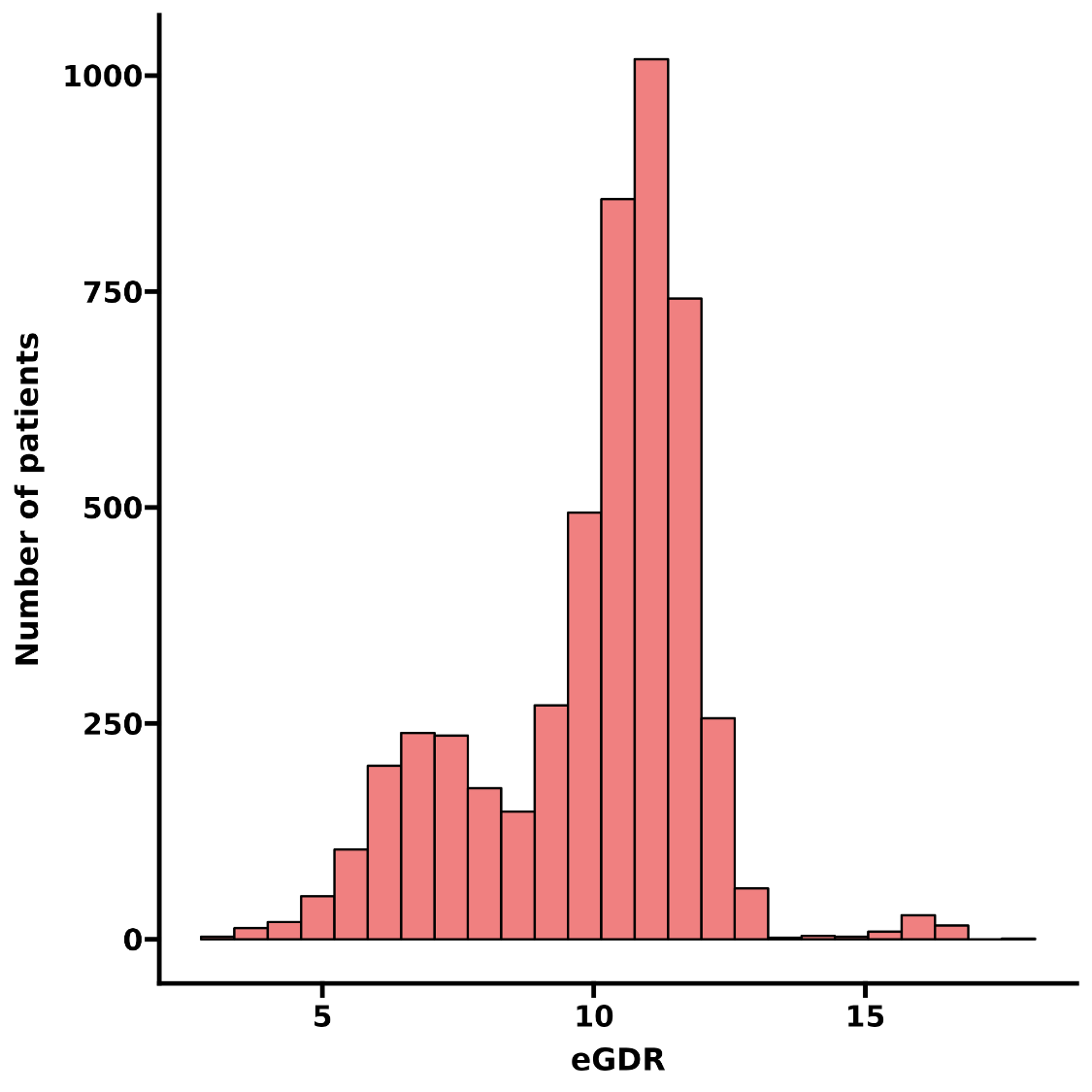
**

**Supplementary Fig. 2** The distributions of the eGDR index for **(A)** CVD, **(B)** heart disease, and **(C)** stroke, were analyzed to assess its association with these outcomes. It shows how the eGDR values differ between individuals with and without these conditions. Abbreviations: *eGDR*, estimated glucose disposal rate; *CVD*, cardiovascular disease

**
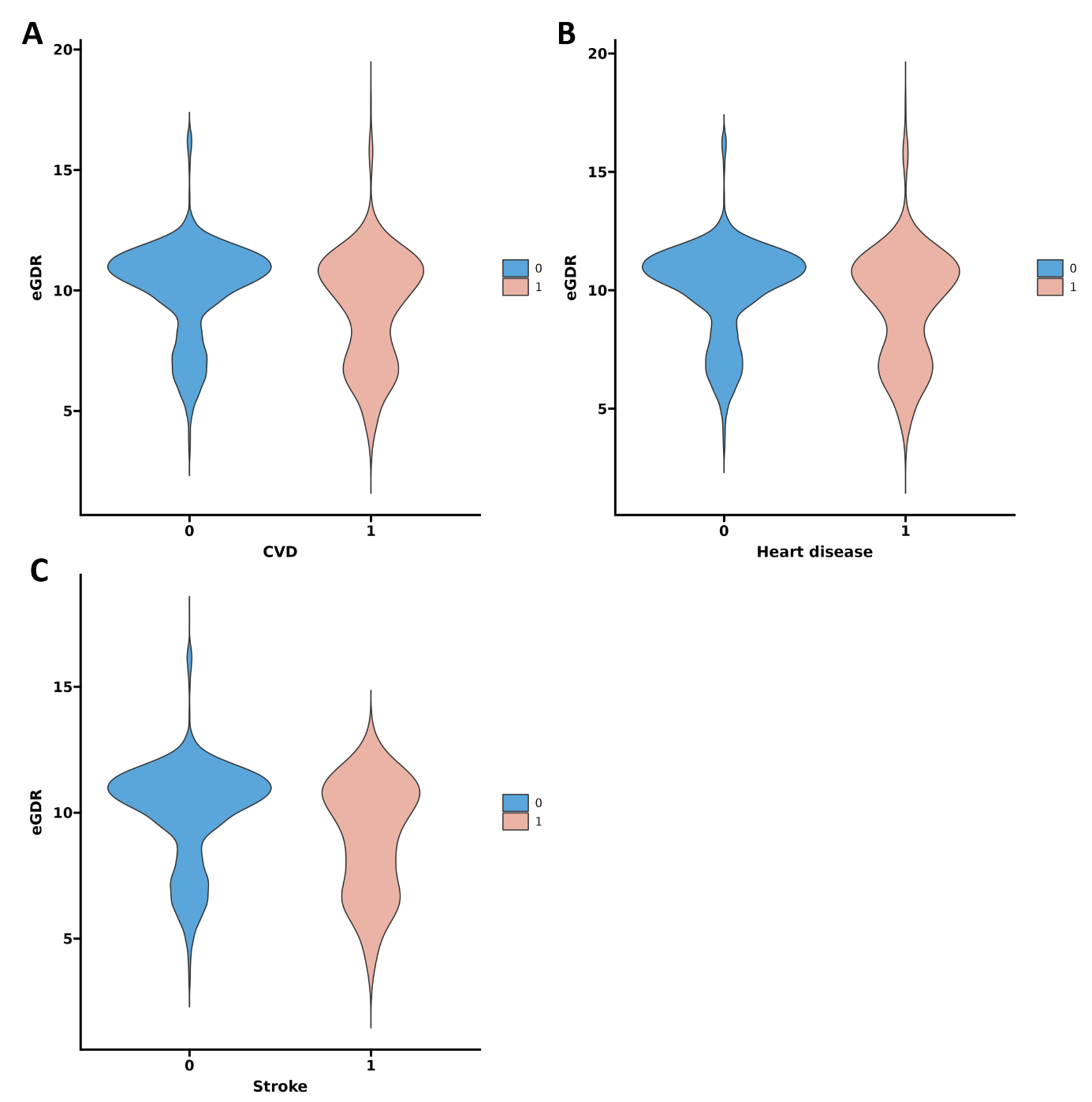
**

**Supplementary Fig. 3** Spearman’s correlations among the baseline clinical data. Abbreviations: *WC*, waist circumference; *BMI*, body mass index; *eGDR*, estimated glucose disposal rate; *CVD*, cardiovascular disease

**
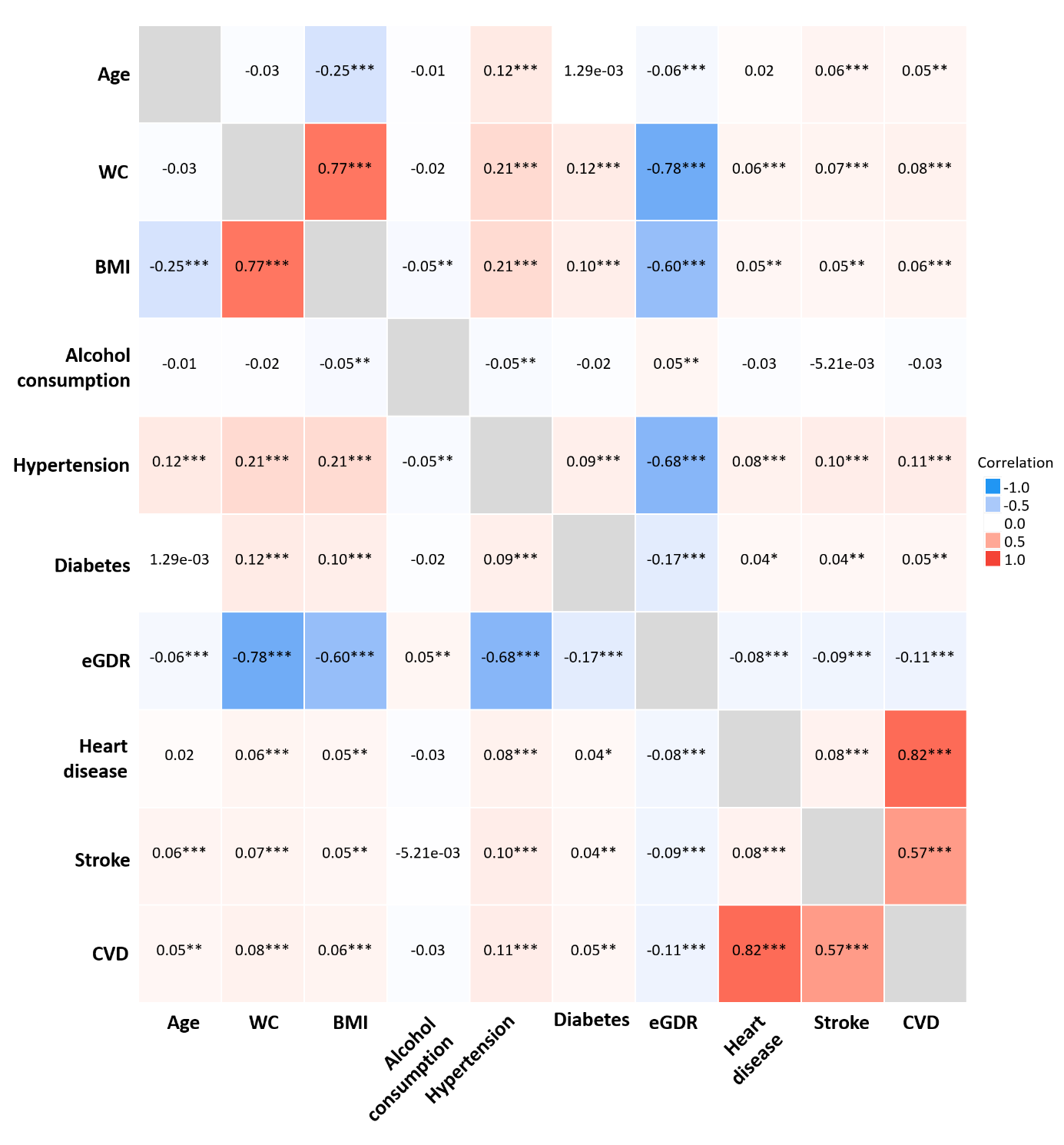
**

**Supplementary Fig. 4** The distributions of the six variables (age, BMI, WC, hypertension, diabetes, and alcohol consumption status) selected for developing the basic predictive model for **(A)** CVD, **(B)** heart disease, and **(C)** stroke. Abbreviations: *CVD*, cardiovascular disease; *BMI*, body mass index; *WC*, waist circumference


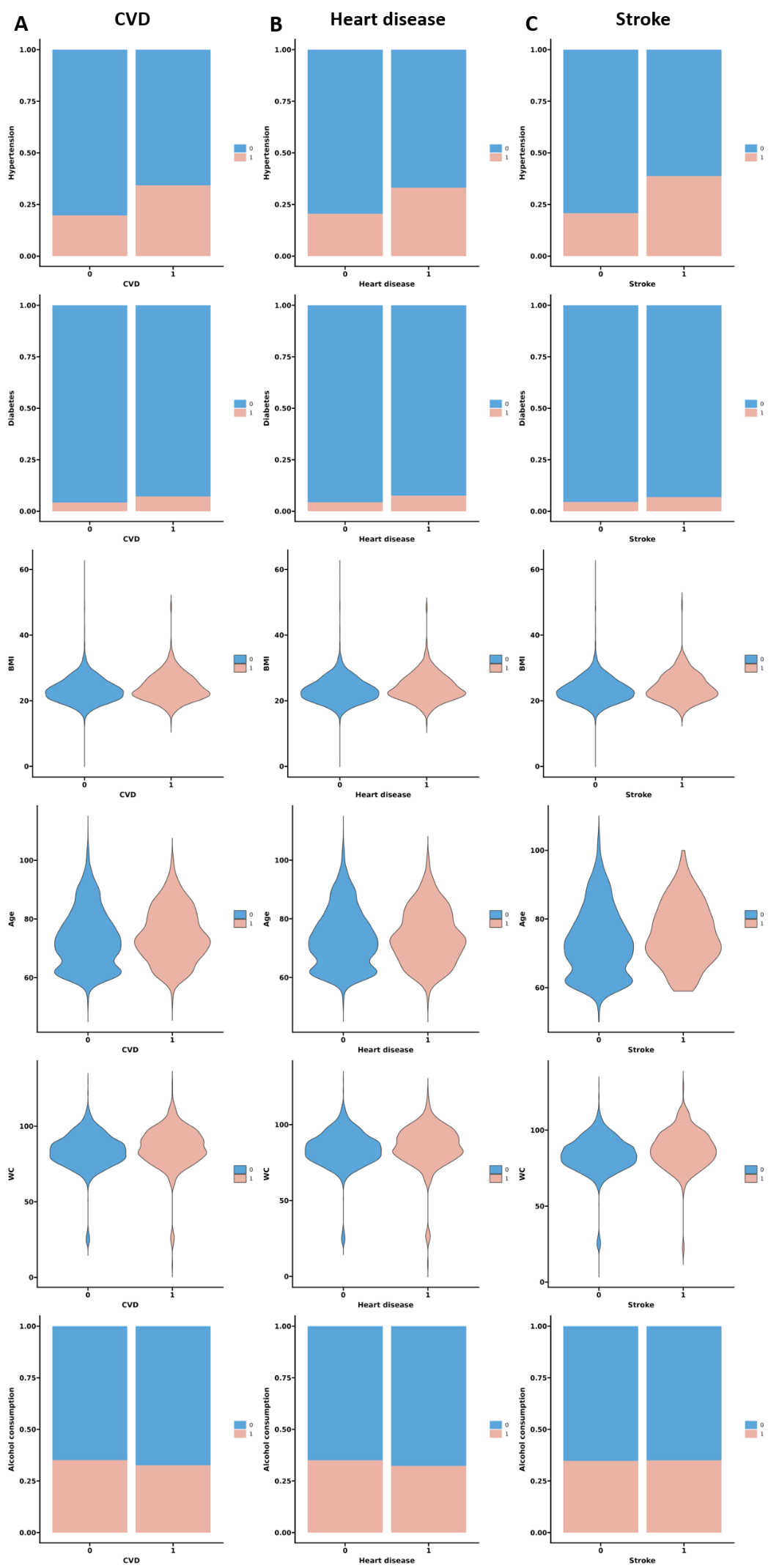


**Supplementary Fig. 5** DCA and calibration curves of the modified ML model, which incorporated the estimated glucose disposal rate, were evaluated for predicting CVD, heart disease, and stroke in both the training and testing sets. **(A-F)** DCA curves for predicting CVD, heart disease, and stroke in both the training and testing sets. **(G-L)** Calibration curves for predicting CVD, heart disease, and stroke in both the training and testing sets. Abbreviations: *DCA*, decision curve analysis; *ML*, machine learning; *CVD*, cardiovascular disease


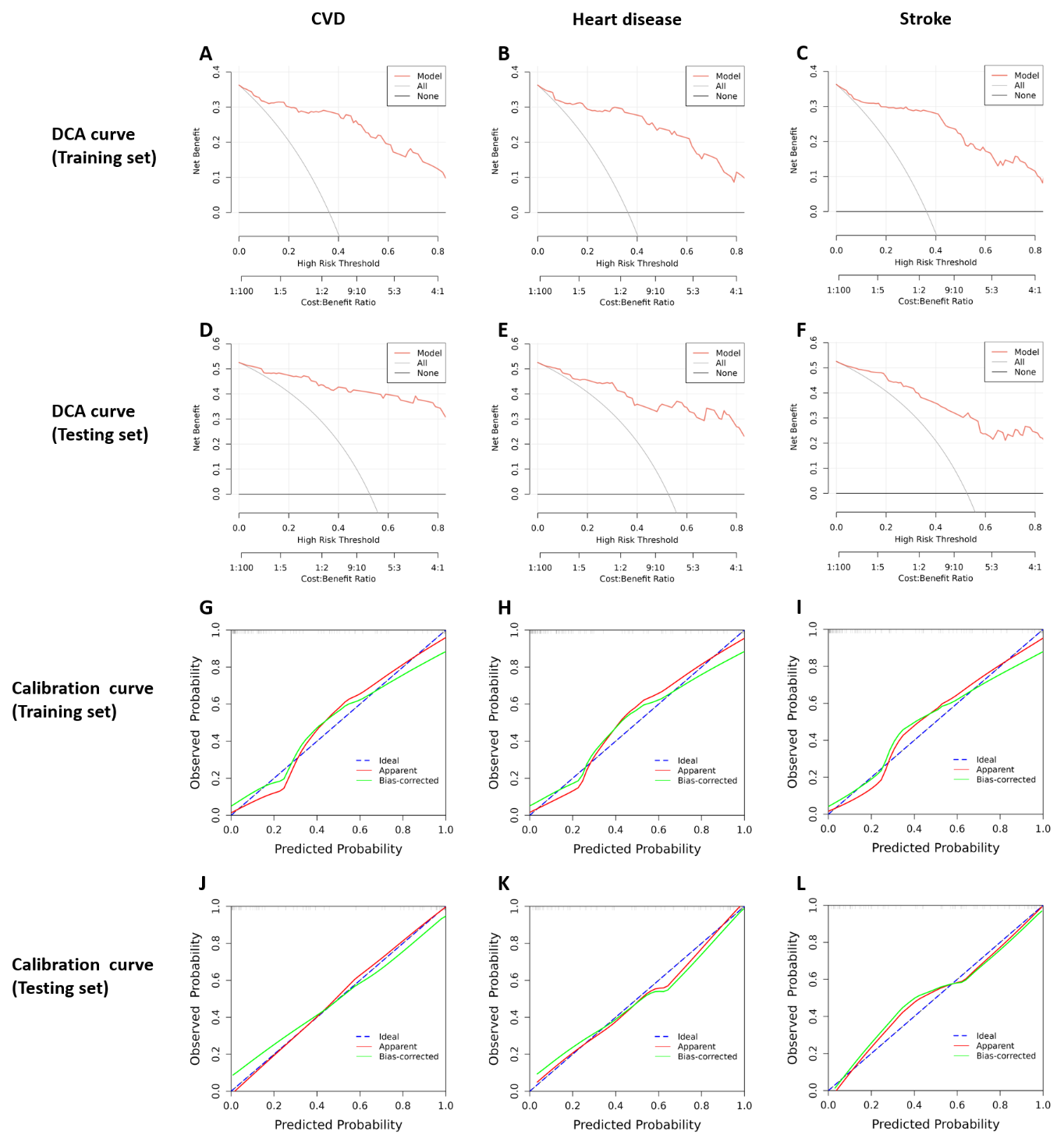

Supplement: Supplementary file 1 — Supplementary Material 1. [file 12933_2025_2729_MOESM1_ESM.docx]
